# Uncovering the specificity and predictability of tryptophan metabolism in lactic acid bacteria with genomics and metabolomics

**DOI:** 10.3389/fcimb.2023.1154346

**Published:** 2023-03-13

**Authors:** Tong Pan, Zhangming Pei, Zhifeng Fang, Hongchao Wang, Jinlin Zhu, Hao Zhang, Jianxin Zhao, Wei Chen, Wenwei Lu

**Affiliations:** ^1^ State Key Laboratory of Food Science and Technology, Jiangnan University, Wuxi, China; ^2^ School of Food Science and Technology, Jiangnan University, Wuxi, China; ^3^ School of Food Science and Technology, Shihezi University, Shihezi, China; ^4^ National Engineering Research Center for Functional Food, Jiangnan University, Wuxi, China; ^5^ (Yangzhou) Institute of Food Biotechnology, Jiangnan University, Yangzhou, China; ^6^ International Joint Research Laboratory for Pharmabiotics & Antibiotic Resistance, Jiangnan University, Wuxi, China

**Keywords:** tryptophan, metabolism, lactic acid bacteria, specificity, predictability, genomics, metabolomics

## Abstract

Tryptophan is metabolized by microorganisms into various indole derivatives that have been proven to alleviate diseases and promote human health. Lactic acid bacteria (LAB) are a broad microbial concept, some of which have been developed as probiotics. However, the capacity of most LAB to metabolize tryptophan is unknown. In this study, the aim is to reveal the rule of tryptophan metabolism in LAB by multi-omics. The findings showed that LAB were rich in genes for tryptophan catabolism and that multiple genes were shared among LAB species. Although the number of their homologous sequences was different, they could still form the same metabolic enzyme system. The metabolomic analysis revealed that LAB were capable of producing a variety of metabolites. Strains belonging to the same species can produce the same metabolites and have similar yields. A few strains showed strain-specificity in the production of indole-3-lactic acid (ILA), indole-3-acetic acid, and 3-indolealdehyde (IAld). In the genotype-phenotype association analysis, the metabolites of LAB were found to be highly consistent with the outcomes of gene prediction, particularly ILA, indole-3-propionic acid, and indole-3-pyruvic acid. The overall prediction accuracy was more than 87% on average, which indicated the predictability of tryptophan metabolites of LAB. Additionally, genes influenced the concentration of metabolites. The levels of ILA and IAld were significantly correlated with the numbers of aromatic amino acid aminotransferase and amidase, respectively. The unique indolelactate dehydrogenase in *Ligilactobacillus salivarius* was the primary factor contributing to its large production of ILA. In summary, we demonstrated the gene distribution and production level of tryptophan metabolism in LAB and explored the correlation between genes and phenotypes. The predictability and specificity of the tryptophan metabolites in LAB were proven. These results provide a novel genomic method for the discovery of LAB with tryptophan metabolism potential and offer experimental data for probiotics that produce specific tryptophan metabolites.

## 1 Introduction

Tryptophan metabolites play an important role in both the development of diseases and the homeostasis of the human body’s internal environment (Platten et al., 2019). The catabolism of tryptophan in mammals mainly includes the kynurenine (KYN) pathway, the serotonin pathway, and the indole derivative pathway, of which the indole derivatives can only be produced by microbes (Agus et al., 2018). The KYN pathway is the main direction of tryptophan catabolism, which consumes about 90% of tryptophan (Cervenka et al., 2017). However, previous studies found that metabolites produced along the KYN pathway can help tumors escape immune surveillance and promote their development (Offringa et al., 2022). Tryptophan can be metabolized by microorganisms into a variety of indole derivatives, which the host is unable to produce. The KYN pathway competes with this metabolic pathway. Recently, an increasing number of studies have demonstrated that indole derivatives played a significant role in preventing the onset and progression of diseases, particularly in chronic conditions like inflammatory bowel disease (Flannigan et al., 2022), atopic dermatitis (Fang et al., 2022), Alzheimer’s disease (Sun et al., 2022), alcoholic liver disease (Wrzosek et al., 2021), and coronary artery disease(Li et al., 2022). Intestinal microorganisms are the key to produce indole derivatives that can alleviate chronic diseases. *In vitro* fermentation experiments showed that *Clostridium* and *Peptostreptococcus* could produce 3-indoleacrylic acid (IA) and indole-3-propionic acid (IPA) (Dodd et al., 2017; Wlodarska et al., 2017). IA was thought to ease colitis and improve intestinal epithelial barrier performance. The risk and severity of atherosclerosis were significantly negatively correlated with the serum IPA concentration in patients with coronary heart disease (Xue et al., 2022). *Bifidobacterium longum* could produce 3-indolealdehyde (IAld) to relieve symptoms of patients with atopic dermatitis by activating AhR (Fang et al., 2022). *Bifidobacterium* has also been proven to metabolize tryptophan to indole-3-lactic acid (ILA), which could help build the infant’s early immune system (Laursen et al., 2021). However, ILA, IAld and indole-3-acetic acid (IAA) could be metabolized by multiple microorganisms, such as *Escherichia coli*, *Bacteroides*, *Clostridium*, and *Faecalibacterium prausnitzii* (Roager and Licht, 2018). To sum up, these intestinal microorganisms jointly regulate the complex metabolism of indole derivatives and have a profound impact on human health. Therefore, regulating the metabolism of indole derivatives by intestinal microorganisms to promote human health has become the research hotspot.

Lactic acid bacteria (LAB) are a class of bacteria that can ferment and produce lactic acid. Some LAB are considered to have prebiotic functions and have been developed as dietary supplements to be used to regulate the balance of the human intestinal environment and promote the beneficial shift of gut microbiota (De Filippis et al., 2020). Previous studies demonstrated some LAB can convert tryptophan into indole derivatives and influence human immunity (Zelante et al., 2013; Cervantes-Barragan et al., 2017). However, currently known LAB that can metabolize tryptophan are mainly *Limosilactobacillus reuteri*, and the metabolic ability of other species is unclear. Previous studies have shown that *Bifidobacterium* of the same species but from different sources have different abilities to use carbon sources (Liu et al., 2021). Therefore, it is also necessary to investigate whether the isolation source has an impact on the LAB’ capacity to metabolize tryptophan. The evidence indicated that a complete metabolic enzyme system can predict any metabolite of microorganisms (Rath et al., 2017; Heinken et al., 2019). The necessity of the tryptophan metabolism gene in the transformation of indole derivatives by microorganisms has been confirmed (Hutcheson and Kosuge, 1985; Koga, 1995; Williams et al., 2014; Dodd et al., 2017; Wlodarska et al., 2017), and the bacterial tryptophan metabolism profile, jointly constructed by catabolites and enzymes, has also been improved (Roager and Licht, 2018). Therefore, it seems possible to use comparative genomic approaches to construct tryptophan metabolism enzyme spectra of different LAB and explain the tryptophan metabolism ability of LAB through association analysis with metabolomics.

In this study, we selected 148 strains from 13 LAB species. These species have been extensively studied and were considered beneficial to human wellness. Some species appeared to be involved in tryptophan metabolism. For instance, it has been demonstrated that *L. reuteri* DSM 20016 produced IAld and ILA (Zelante et al., 2013). However, in-depth research has not been reported to determine whether this metabolic capability is universal in *L. reuteri*. Therefore, in order to explore the specificity of tryptophan metabolism in these species and their prbiotic potential, we conducted the following experiments: The homology of the tryptophan metabolism genes in LAB was analyzed by using comparative genomic approaches, and the potential metabolites were predicted. Additionally, the capacity of every strain to metabolize tryptophan *in vitro* was investigated using targeted metabolomics. Combing genomic analysis with metabolomic results according to bioinformatic methods, we demonstrated the predictability of tryptophan metabolites in LAB. We also explored the effect of the number of genes on the metabolism of tryptophan and found two kinds of indolelactate dehydrogenase that may control the high production of ILA in *Ligilactobacillus salivarius*.These results help to understand the complex metabolism of tryptophan in LAB and provide an experimental basis and new ideas for the development of probiotics.

## 2 Materials and methods

### 2.1 Selection of strains

The 148 strains used in this study were isolated from the Culture Collection of Food Microorganisms of Jiangnan University (Wuxi, China), and we sequenced the genomes of each strain individually. They are distributed in 13 species from 6 genera (**Table 1**). The specific information for all LAB is listed in **Table S1**.

**Table 1:** Lactic acid bacteria speices in this study.

Genus	Species	No. of Strains	Source
*Lacticaseibacillus*	*paracasei*	10	Human feces; Chinese pickle
*Lacticaseibacillus*	*rhamnosus*	9	Human feces
*Lactiplantibacillus*	*plantarum*	10	Human feces; Chinese pickle
*Lactiplantibacillus*	*pentosus*	8	Human feces; pickles
*Lactobacillus*	*acidophilus*	8	Human feces
*Lactobacillus*	*crispatus*	14	Human feces; Human vagina
*Lactobacillus*	*gasseri*	8	Human feces
*Lactobacillus*	*helveticus*	9	Fermented yak milk; Yak qula; Dairy fan
*Latilactobacillus*	*curvatus*	4	Human feces
*Ligilactobacillus*	*salivarius*	12	Human feces
*Limosilactobacillus*	*fermentum*	11	Human feces
*Limosilactobacillus*	*mucosae*	15	Human feces
*Limosilactobacillus*	*reuteri*	30	Fermented rice milk; Human feces

### 2.2 DNA extraction, sequencing, and prediction of coding sequences

All LAB were cultured on MRS plates for 24 hours; single colonies were selected and cultured in MRS broth for 12–14 hours until the early stage of stabilization. The culture was removed after centrifugation at 5000 x g for 10 min, and bacterial cells were washed with 0.9% sterile saline and collected under the same centrifugation conditions. The DNA of LAB was extracted according to the operation process of the rapid bacterial genetic DNA isolation kit (Sangon Biotech Ltd., Shanghai, China).

Illumina HiSeq platform (Novogene Biotech Ltd., Tianjin, China; Majorbio Biotech Ltd., Shanghai, China), which produced 2 × 150-bp pair-end read libraries, was used for genome sequencing. For each sample, the raw data were provided and then trimmed into high-quality reads with a minimum of 100 genome coverage depth (clean data). The software SOAPdenovo2 (Luo et al., 2012) was used to assemble contigs, and then we tested various Kmer values and obtained the optimal assembly result. The assembly result was then partially assembled and optimized to form scaffolds based on the relationship between paired-end reads and read overlaps.

The software Prodigal (version, 2.6.3; -p, single; -g, 11) predicted the coding sequences in all LAB genomes and translated them into protein sequences for subsequent homologous protein searching (Hyatt et al., 2010).

### 2.3 Identification of genes associated with tryptophan metabolism

We have identified the genes involved in microbial tryptophan metabolism and the corresponding Enzyme Commission (EC) number by consulting previous studies on microbial tryptophan metabolism (Agus et al., 2018; Roager and Licht, 2018) and the gene information displayed in the tryptophan metabolism pathway in Kyoto Encyclopedia of Genes and Genomes (KEGG, https://www.kegg.jp). The specific information on tryptophan metabolic enzymes involved in this study is listed in **Table S2**. We obtained the protein sequence corresponding to the tryptophan metabolism gene from the National Center for Biotechnology Information (NCBI, https://www.ncbi.nlm.nih.gov) RefSeq and GeneBank databases by the full name of the enzyme and EC number. The tryptophan metabolic enzymes of LAB were identified using these proteins as reference sequences.

### 2.4 Homology search and prediction of tryptophan metabolites

The homology analysis of LAB proteome was completed by DIAMOND (version, 2.0.14) BLASTP (identity, 30%; E value, 1e-3; subject cover, 70%; query cover, 70%; –ultra-sensitive) (Buchfink et al., 2021). The specific homology search information for all strains is listed in **Table S3**. Hit sequences that satisfy the aforementioned parameters are regarded as homologous sequences involved in the metabolism of tryptophan. The sequences with the highest homology were used to summarize the tryptophan metabolic enzyme results in LAB, and each strain’s tryptophan metabolic products were predicted. Strains with a complete enzyme system can produce the corresponding metabolites. For instance, it is predicted that a strain will be able to produce indole propionic acid if it possesses the homologous sequences of aromatic amino acid aminotransferase (ArAT), indolelactate dehydrogenase (*fldH*) or lactate dehydrogenase (LDH), cinnamoyl-coA: chenyllactate coA-transferase (*fldA*), phenyllactoyl-coA dehydratase alpha/beta (*fldBC*), R-phenyllactate dehydratase activator (*fldI*), and phenylacrylate reductase (*acdA*).

### 2.5 Extraction of tryptophan metabolites from *in vitro* fermentation

Strains from glycerol preservation tubes were transplanted to MRS plates and grew at 37°C until a single colony appeared. Single colonies were then selected and grown in MRS broth for 12 hours to the late logarithmic phase (two groups of culture media, one for further experiments and the other for determining CFU/OD). ensuring that there are 10^9^ CFU of bacteria in total per milliliter. Bacterial precipitation is washed twice with physiological saline after MRS broth has been centrifuged at 4000 x g for 10 min. According to previous studies, there was a significant negative correlation between microorganisms’ capacity to metabolize amino acids and their rate of growth. These catabolic reactions need to be carried out effectively under the condition of non-rapid growth of bacteria (Vieira-Silva et al., 2016). Therefore, resting cell fermentation (Dodd et al., 2017) was used to explore the tryptophan metabolism ability of LAB. Briefly, after 1 h of resuspended bacterial precipitation in potassium phosphate buffer, the ATP level reaches a constant value, indicating that the majority of the remaining substrate has been consumed (Johann Bader, 1983). The bacteria cells were separated by centrifugation and resuspended in buffer containing 1 mM tryptophan, which was similar to the concentration of tryptophan in the small intestine (Koper et al., 2022). After one hour of standing at 37°C, the supernatant needed to be immediately frozen at -80°C for future metabolomic analysis.

### 2.6 Metabolomics

The LAB *in vitro* fermentation supernatant samples undergone the following pretreatments: First, 100 μL of fermentation supernatant and 400 μL of pre-cooled methanol were fully mixed, and then stood at -20°C for 30 min. To remove the protein, the mixture was centrifuged at 20000 × g for 15 min. After that, 300 μL of the supernatant was vacuum-dried at 45°C. The obtained dry matter was resuspended in 100 μL of methanol dilution (water: methanol=4:1) and filtered through the 0.22 μm microporous membrane. The filtrate was tested for tryptophan metabolites using ultra-high performance liquid chromatography-mass spectrometry (UHPLC-MS).

Compounds were separated by using ACQUIRE UPLC BEH C18 column (Waters, Milford, MA, USA; 1.7 m, 2.1 100 mm) and detected by using Vanquish UHPLC Q-Exactive Plus MS (Thermo Fisher, CA, USA) with extended a dynamic range (100-300 m/z) in positive ion scanning mode. The mobile phase consisted of eluent A (acetonitrile, Supelco, Sigma-Aldrich) and eluent B (0.1% formic acid, Supelco, Sigma-Aldrich). The sample with a volume of 2 μL was injected into the mobile phase, and chromatographic separation was performed at a flow rate of 0.3 mL/min. The linear gradient of the mobile phase was as follows: 5% A and 95% B in the initial 3 min; 3–9 min, eluent A from 5% to 30%, eluent B from 95% to 70%; 9–15 min, mobile phase A rising from 30% to 100%; kept 100% mobile phase A unchanged for 15-16.5 min; in the last 3.5 min, maintained 5% mobile phase A and 95% mobile phase B for rebalancing the column. The indole derivatives metabolized by LAB were qualitatively and quantitatively analyzed using a standard curve. The metabolism of all strains has been listed in **Table S4**. The specific information, such as the retention time of reference materials, has been listed in **Table S5**.

### 2.7 Phylogenetic analysis

To identify the potential key players in the production of ILA, the homologous protein sequences annotated as *fldH* and without specific substrate catalytic function were used to constructed the phylogenetic tree. The phylogenetic tree of LDH was also built. MUSCLE (version, 3.8.31) was used to align all homologous sequences of *fldH* and LDH respectively (Edgar, 2004) and IQ-TREE (version 2.2.0-Linux; model, MF; bootstrap, 1000) was used to build the evolutionary tree of the sequence(Nguyen et al., 2015). iTOL is used to visualize the outcomes(Letunic and Bork, 2021). Most branches of the phylogenetic tree of *fldH* and LDH were folded to better illustrate the evolutionary distance between the unique protein sequence and other sequences, but this did not modify the topological structure of the phylogenetic tree.

### 2.8 Protein structure analysis

AlphaFold2 (version, 1.4; pair mode, unpaired_paired; model type, default) was used to construct the *fldH* homologous protein in *L. salivarius* FWXBH185 and the experimentally verified *fldH* (Uniprot: J7SHB8) protein structure in *C. sporogenes* ATCC 15579. We used the Pairwise Structure Alignment function (algorithm, jFATCAT-rigid) provided by PDB (https://www.rcsb.org) to establish residue-residue correspondence between *L. salivarius* FWXBH185 *fldH* and *C. sporogenes* ATCC 15579 *fldH*.

### 2.9 Statistical analysis and visualization

IBM SPSS Statistics 26 (SPSS Inc., Chicago, IL, USA) was used to calculate Spearman’s rank correlation coefficient (Spearman’s ρ; Benj n Hochberg false discovery rate [FDR]). The Mann-Whitney test is used to analyze whether there are metabolic differences among strains belonging to the same species but from different sources. All data sets were collated using Python 3. Graphpad Prism 9 (La Jolla, California, United States) was used for data visualization. The subsequent editing of all figures was completed with Adobe Illustrator CC2022.

## 3 Results

### 3.1 LAB are rich in genes of tryptophan catabolism

**Figure 1:**
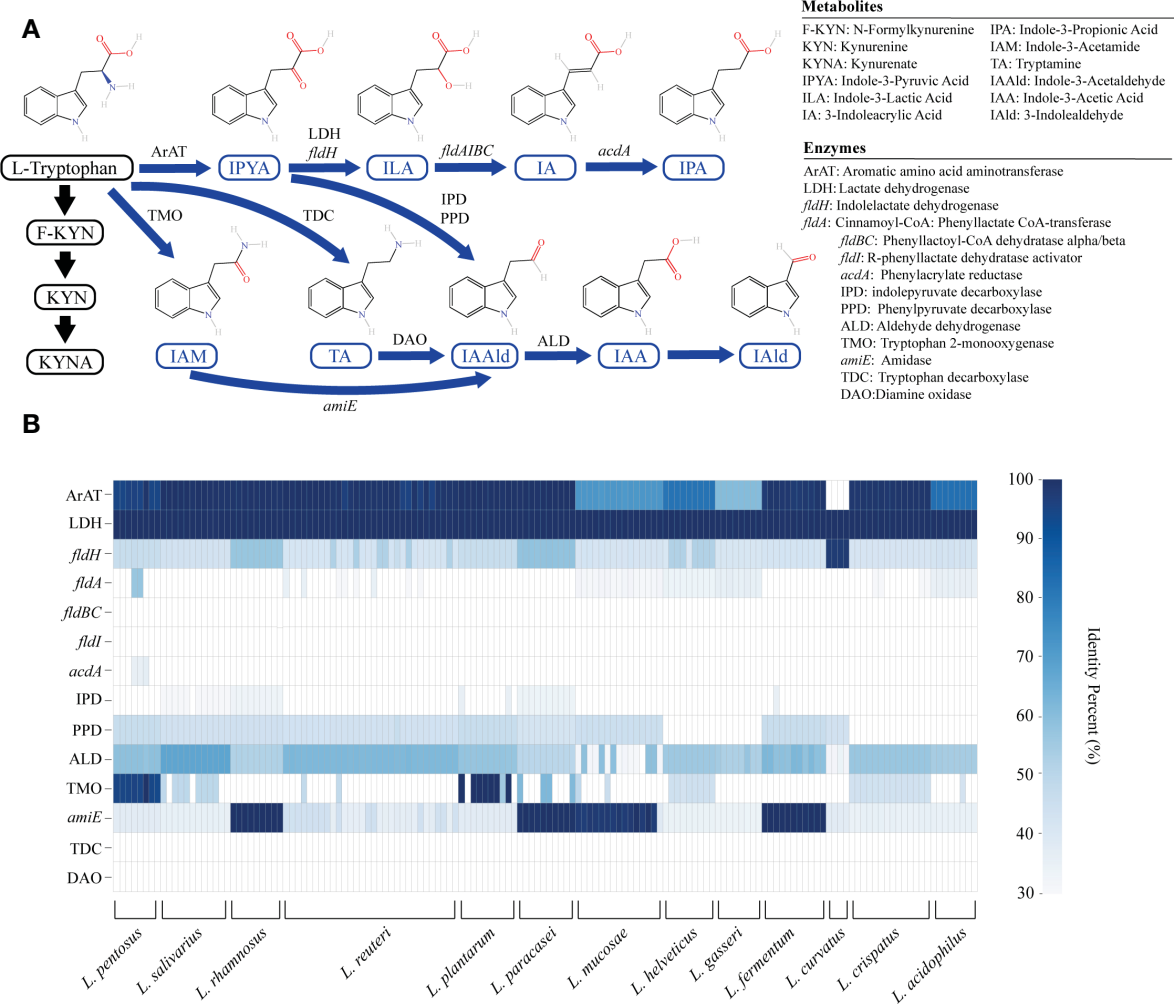
The genome of lactic acid bacteria (LAB) is rich in tryptophan metabolism genes. **(A)** The primary tryptophan metabolites and metabolic network. The host pathway is represented by black, and the microbial pathway is represented by blue. **(B)** The heatmap represents the sequence identity of the best hit of the tryptophan metabolism genes in LAB strains. Genes with more than 30% identity are considered homologous sequences.

The KYN pathway, the indole pathway, and the serotonin pathway are the three main metabolic pathways for tryptophan. Only microbes are capable of producing indole derivatives. In this study, we focused on nine indole derivatives that play an important role in human health. Except for the enzyme that converts IAA into IAld, the metabolic enzyme spectrum of other indole derivatives is clear (**Figure 1A**).

**Figure 2:**
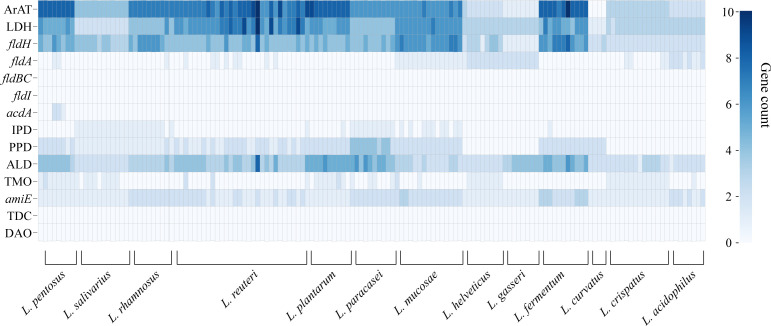
The heatmap shows the number of tryptophan metabolism genes contained in lactic acid bacteria strains, and the color depth indicates the homologous tryptophan metabolism gene count.

We annotated the tryptophan metabolic genes of 148 strains (**Table 1**) to determine whether LAB encode these tryptophan metabolic enzymes (**Table S3**). As a result of LAB metabolic genome annotation, the sequence with the highest homology was displayed (**Figure 1B**), and all homologous sequences were in the corresponding gene count (**Figure 2**). ArAT, Tryptophan decarboxylase (TDC), and Tryptophan 2-monooxygenase (TMO) are three enzymes that directly use tryptophan as a substrate (**Figure 1A**). We found that all strains did not encode TDC, and the tryptamine (TA) metabolic pathway did not exist in LAB. The majority of strains also failed to locate the TMO homologous sequence, but all LAB contained amidase (*amiE*). Except for *Latilactobacillus curvatus*, ArAT was found in the genome of all LAB, and most (10/12 species) LAB contained multiple ArAT (**Figure 2**), which was reported that existed widely in *L. reuteri* previously (Ozcam et al., 2019). Tryptophan will be converted to ILA by *fldH* or LDH after being catabolized to indole-3-pyruvic acid (IPYA) by ArAT (Dodd et al., 2017; Laursen et al., 2021). The evidence of *fldH* was first found in *Clostridium*, and the previous labeling of *fldH* in *Lactobacillus* was mostly replaced by LDH (Montgomery et al., 2022), which was enriched in LAB, especially in *Limosillactobacillus* (**Figure 2**). LDH and *fldH* originate from the same EC number (1.1.1.-), and the protein sequence homology was ~40%. In this study, most of the homologous proteins of *fldH* in LAB were annotated as D-2-hydroxyacid dehydrogenase (HdhD) in NCBI, which were also from the same EC number as *fldH*, and the sequence homology was ~40%. This might indicate that HdhD is a neglected ILA dehydrogenase. In addition, the directly adjacent of the *fldH* and ArAT in some LAB suggested that they are functional metabolic operons (**Table S3**). In terms of genotype, LAB other than *L. curvatus* appeared to be able to produce ILA, but none of them created a gene bridge for the transformation of ILA into IA because they were all deficient in the *fldIBC* functional gene cluster. All LAB contained aldehyde dehydrogenase (ALD), just like ArAT. Additionally, some strains encoded indolepyruvate decarboxylase (IPD) or phenylpyruvate decarboxylase (PPD), which together contributed to the conversion of tryptophan to indoleacetic acid.

The tryptophan metabolites of LAB were then predicted using the gene annotation results (**Table 2**). Since *L. curvatus* contained no first-step metabolic enzyme, it was considered that it did not have any metabolic ability for indole derivatives. Other LAB maintained a high degree of similarity in the production of IPYA, TA, ILA, IA, and IPA. The predicted results of other metabolites were species- or strain-specific. To sum up, LAB encoded a large number of tryptophan metabolic genes. LAB had genetic evidence for the metabolism of tryptophan into multiple metabolites.

**Table 2:** Predicted tryptophan metabolites of lactic acid bacteria at the species level.

Species	No. of Strains	Proportion of strains in the species that could produce the following metabolites (%)							
		IPYA	ILA	IA	IPA	IAM	TA	IAAld	IAA
*L. acidophilus*	8	100	100	0	0	12.5	0	0	12.5
*L. crispatus*	14	100	100	0	0	100	0	0	100
*L. curvatus*	4	0	0	0	0	0	0	0	0
*L. fermentum*	11	100	100	0	0	0	0	100	100
*L. gasseri*	8	100	100	0	0	0	0	0	0
*L. helveticus*	9	100	100	0	0	88.9	0	0	88.9
*L. mucosae*	15	100	100	0	0	13.3	0	100	100
*L. paracasei*	10	100	100	0	0	40	0	100	100
*L. plantarum*	10	100	100	0	0	80	0	100	100
*L. reuteri*	30	100	100	0	0	6.7	0	100	100
*L. rhamnosus*	9	100	100	0	0	0	0	100	100
*L. salivarius*	12	100	100	0	0	66.7	0	100	100
*L. pentosus*	8	100	100	0	0	100	0	100	100

### 3.2 The specificity of LAB in tryptophan metabolites

**Figure 3:**
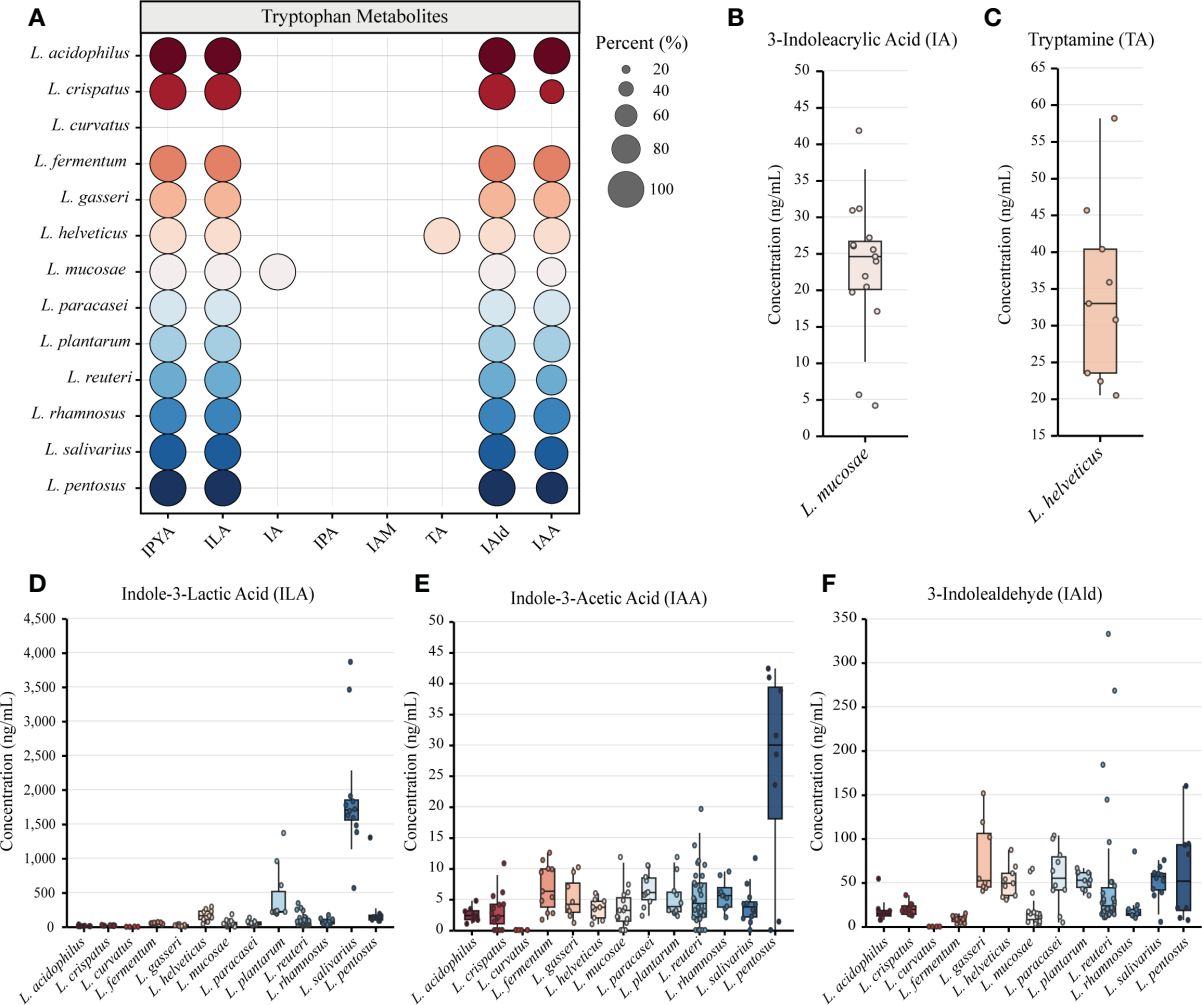
Metabolomics revealed the tryptophan metabolism ability of lactic acid bacteria (LAB). **(A)** LAB species metabolize a variety of indole derivatives. The proportion of strains within a species that produce metabolites is indicated by the size of the circle. Different colors represent different species. **(B–F)** The concentrations of all tryptophan metabolites detected in the fermentation broth of different strains are shown by box plots. All data were collected by repeating three experiments and taking the average of the results. Tukey’s honestly significant difference test was used to exclude discrete points.

We performed *in vitro* experiments to confirm the outcomes of gene prediction after obtaining genomic evidence of tryptophan metabolism in LAB. The findings demonstrated that LAB could generate a wide range of tryptophan metabolites, and the majority of LAB maintained a high level of species specificity in metabolism (**Figure 3A**). IA (4.139-41.789 ng/mL) was found in the fermentation broth of all strains of *Limosillactobacillus mucosae* (**Figure 3B**), but prior genomic homology analysis had not revealed any homologous sequences with the *fldIBC* gene cluster. No strain can produce IPA, which is consistent with the result of gene prediction. However, tryptamine (20.454-58.145 ng/mL) was detected in the fermentation of all strains of *Lactobacillus helveticus* (**Figure 3C**), which, like IA, also lacked genetic evidence. The production of ILA demonstrated that IPYA had appeared in the fermentation process because IPYA was the only upstream product of ILA, even though we were unable to detect IPYA in the fermentation supernatant of LAB. IPYA might be rapidly converted into ILA or indole-3-acetaldehyde (IAAld), which made it difficult to enrich. As a result, we believed that LAB, except for *L. curvatus*, were capable of producing IPYA and ILA, which is consistent with the findings of gene prediction. Additionally, compared with other LAB (0-1368.567 ng/mL, mean = 102.554 ng/mL), *L. salivarius* (566.553-3864.553 ng/mL, mean = 1907.063ng/mL) had a stronger ability to metabolize ILA, especially *L. salivarius* FWXBH185 (3864.553 ng/mL) and FBJSY202 (3463.081 ng/mL) (**Figure 3D**). Contrary to gene prediction results, indole-3-acetamide (IAM) could not be found in all LAB fermentation broths. Its downstream metabolite, IAA, had inconsistent *in vitro* fermentation and gene prediction results, and metabolic concentration varied between species. *Lactiplantibacillus pentosus* (0-40.967 ng/mL, mean = 22.507 ng/mL) produced more IAA than other LAB (0-19.667 ng/mL, mean = 4.594 ng/mL) (**Figure 3E**). We also detected the concentration of IAld in the LAB fermentation broth. Except for *L. curvatus*, other LAB (2.984-332.681 ng/mL, mean = 40.266 ng/mL) could produce IAld, and the yield was relatively conservative at the species level, but some strains of *L. reuteri* had strong IAld metabolism abilities (**Figure 3F**), such as DYNDL2M15 (184.103 ng/mL), DYNDL8M31 (332.681 ng/mL), and FSCPS76L4 (268.246 ng/mL).

We also examined how the tryptophan metabolism of strains from various sources varied. All strains from the same species in this study had the same category of tryptophan metabolites, despite the fact that some species had multiple sources for their isolation (**Table S4**). The yield of ILA of *L. helveticus* DYNDL451 from dairy fan was lower when compared to the strains isolated from fenmented yak milk and yak qula. The results of Mann-Whitney test showed that the IAld metabolic level of *L. reuteri* from fermented rice milk was significantly higher than that of the strain isolated from human feces. There was no significant difference in the tryptophan metabolism level of strains from different sources in other species (**Figure S1**).

### 3.3 Accuracy of prediction by genomic-metabolomic association analysis

**Figure 4:**
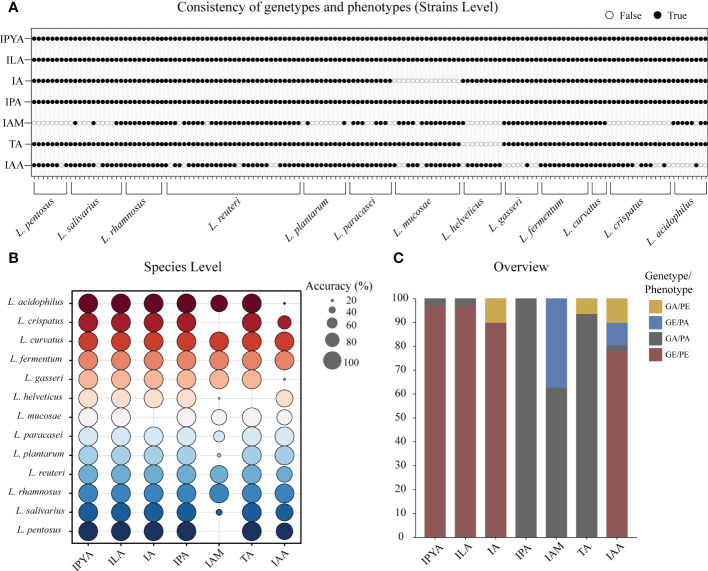
The metabolites of lactic acid bacteria (LAB) predicted by homologous genes had high consistency with actual production. **(A)** The binary diagram illustrates whether the predicted products and metabolites of each strain are consistent. White filling denotes the opposite of the two results, black filling indicates the predicted results are consistent to the metabolic results. **(B)** The consistency of genotype and phenotype is reflected at the species level. The accuracy of the metabolite prediction within a species increases with the size of the prediction circle. Different colors represent different species. **(C)** The stacked column chart provides an overview of the consistency between the metabolomics-based actual metabolites and the genomics-based predicted metabolites in LAB. Phenotype absence (PA); Phenotype presence (PE); Gene presence (GE); Gene absence (GA).

To confirm the gene prediction, we used the metabolism results of LAB. In LAB, only *L. helveticus*, whose TA metabolic gene and true metabolic level could not correspond (**Figure 4A**). Additionally, *L. mucase* produced IA, although no homologous complete *fldAIBC* gene cluster was found in all (**Figure 4B**). IAM and IAA’s actual metabolisms and gene predictions both revealed species-specificity, and the two metabolites’ gene prediction results were less than 80% accurate. The gene prediction results and metabolism of the three metabolites IPYA, ILA, and IPA in all LAB strains are completely consistent, and the accuracy of gene prediction is 100% (**Figure 4C**). The average accuracy of gene prediction is more than 88%.

### 3.4 Effect of gene diversity on the concentration of tryptophan metabolites in LAB

#### 3.4.1 Gene count

**Figure 5:**
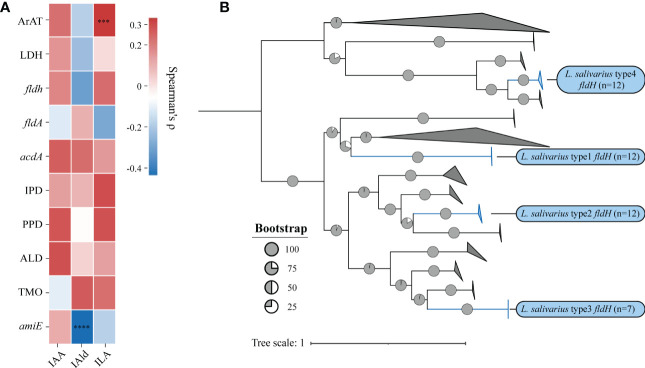
**(A)** The heatmap represents the correlation between the number of genes and the concentration of metabolites, and the statistical significance will be marked only when Spearman’s ρ (Benjamin Hochberg false discovery rate [FDR]) is greater than 0.3.***p = 0.0003, ****p < 0.0001. **(B)** Phylogenetic trees were built using the indolelactate dehydrogenase (*fldH*) homologous sequences of all lactic acid bacteria. Without modifying the topological structure of phylogenetic trees, the folded branches were used to facilitate the display of two different types of *fldH* sequences of *L. salivarius*. The folded cluster of *fldH* from *L. salivarius* was marked blue.

We then investigated the correlation between the quantity of genes and the concentration of metabolites using Spearman’s ρ (**Figure 5A**). The significance would be shown only when the correlation is greater than 0.3. The number of homologous sequences of ArAT was significantly positively correlated with the ILA production of LAB, but Spearman’s ρ was only 0.31, which was a relatively weak correlation. The concentration of IAld was significantly negatively correlated with the number of *amiE*. In conclusion, the homologous genes of tryptophan metabolism found in LAB could be used to predict the ability of LAB to metabolize tryptophan. Additionally, the number of genes may control how metabolites are produced. These findings suggest that genotype and phenotype are strongly correlated.

#### 3.4.2 The IPYA to ILA conversion is primarily controlled by *fldH*

**Figure 6:**
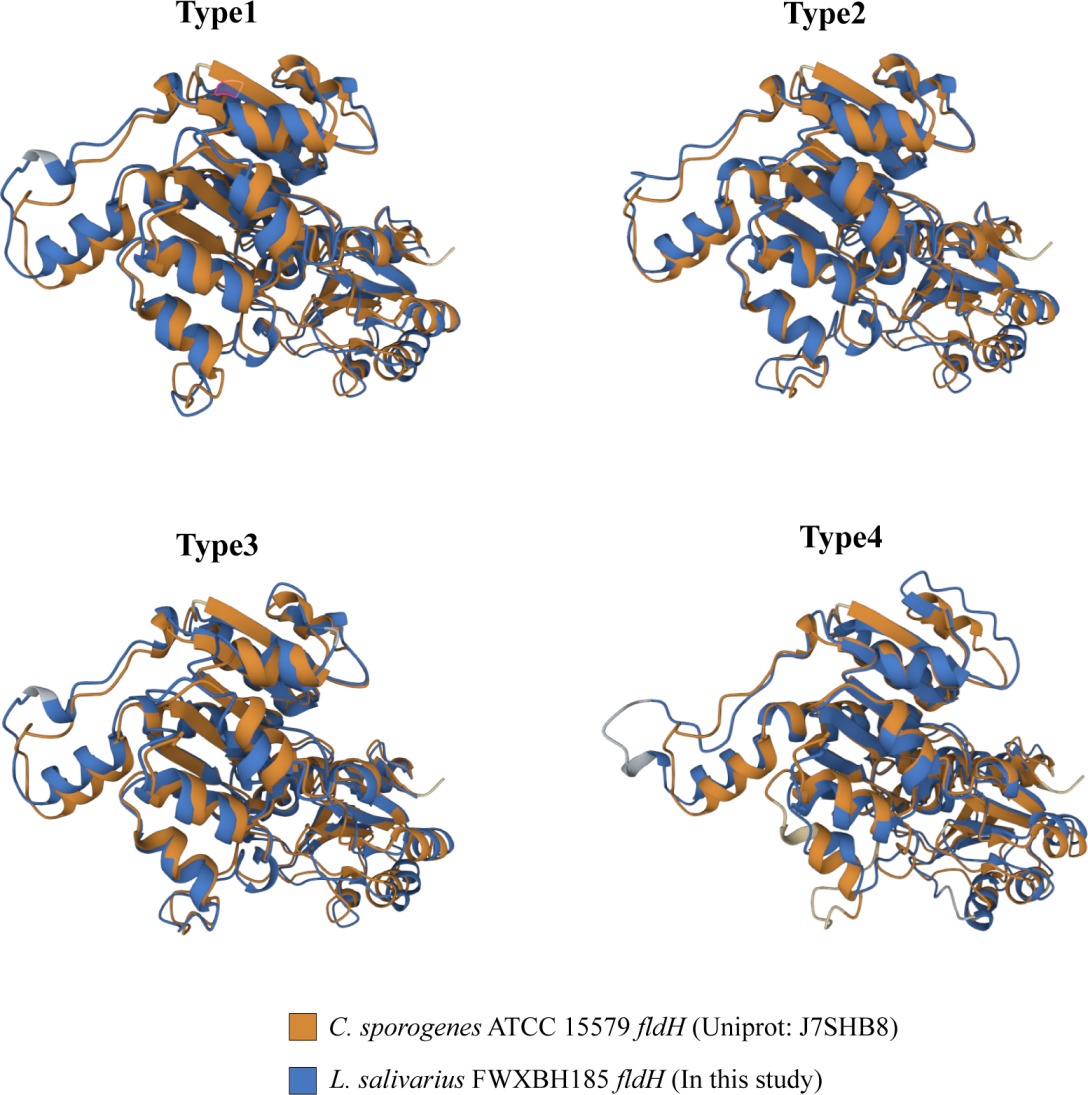
All *fldH* of *L. salivarius* FWXBH185 was compared with the reference *fldH* (Uniprot: J7SHB8) of *C. sporogenes* ATCC 15579 *via* protein structure alignment. The proteins were overlapped to show the similarity of the spatial structure of the two *fldH* proteins. The structure of *C. sporogenes* ATCC 15579 *fldH* was marked with yellow. The *fldH* of *L. salivarius* FWXBH185 was marked with blue, and the type of fldH was marked on the top of the structure.

ILA is currently the most researched indole derivative. It can alleviate the occurrence and development of various diseases and regulate immune homeostasis (Zelante et al., 2013; Cervantes-Barragan et al., 2017; Laursen et al., 2021). In this study, *L. salivarius* demonstrated exceptionally high ILA metabolism capacity (**Figure 3D**). Although the above result showed that the content of ILA was positively correlated with the number of ArAT, the correlation was weak, and the number of ArAT in *L. salivarius* was not the highest among LAB, being far less than that in *Limitsilactobacillus* species. Previous research had shown that a specific LDH controlled ILA production in *Bifidobacterium* (Laursen et al., 2021), whereas *fldH* controlled the metabolic processes of *C. spologenes* ATCC 15579, which could produce a significant amount of ILA at resting cells (Dodd et al., 2017). We, hypothesized that LDH or *fldH* might be the enzyme in charge of regulating ILA production in LAB. The phylogenetic analysis of all *fldH* in LAB revealed that the *fldH* encoded by the *L. salivarius* independently clustered (here called type 1, type 2, type 3, and type 4 *fldH*). Type 4 *fldH* had close genetic distance and high affinity with other LAB. The other three types of *fldH* were far from the fldH of other LAB, suggesting that they may have the unique potential to efficiently convert IPYA into ILA. However, type 1 and type 2 *fldH* could be found in all strains of *L. salivarius*, but type 3 *fldH* only existed in the genomes of 7 strains of *L. salivarius* (**Figure 5B**). The phylogenetic analysis of LDH did not reveal a comparable phenomenon. There are no independent gene clusters, and the genetic distances between LDH of *L. salivarius* and LDH of other LAB were very close (**Figure S2**). Then, we compared the sequence homology, constructed the protein structure and compared the protein structure similarity of the *fldH* (Uniprot: J7SHB8) of *C. spologenes* ATCC 15579 (here called reference *fldH*) and type 1-4 *fldH* of *L. salivarius* FWXBH185, which was the largest production of ILA in all LAB. Type 1, type 2, type 3, and type 4 *fldH* shared 43.6%, 40.6%, 38.5%, and 35.3% homology with reference *fldH*, respectively (**Table 3**). In protein structure alignment, the type 1-3 *fldH* models could well overlap the reference *fldH* protein model (**Figure 6**), which had low root mean square deviation (RMSD) and high template modeling score (TM-score).They might possess the same substrate catalytic center. However, type 4 *fldH* showed poor structural consistency with reference *fldH*, with RMSD ~2.26 and TM-score ~0.85 (**Table 3**).

**Table 3:** The *fldH* structure alignment between *Ligilactobacillus salivarius* FWXBH185 and *Clostridium sporogenes* ATCC 15579.

Type ID	Loci	RMSD	TM-score	Sequence Identity (%)	Reference Coverage (%)	Target Coverage (%)
Type1 *fldH*	Scaffold7_93500 92511	1.71	0.93	43.6	99	99
Type2 *fldH*	Scaffold3_94834 95829	1.82	0.93	40.6	99	99
Type3 *fldH*	Scaffold17_13829 14821	1.85	0.93	38.5	99	99
Type4 *fldH*	Scaffold1_494215 495171	2.26	0.85	35.3	93	97

RSMD, root mean square deviation; TM-score, template modeling score.

## 4 Discussion

The tryptophan metabolism of microorganisms is a complex metabolic network. Numerous enzymes have an impact on the metabolites in this pathway (**Table S2**). To produce the corresponding metabolites, a complete metabolic enzyme system is required (Dodd et al., 2017). Previous studies have found a small amount of gene evidence for tryptophan metabolism-related enzymes encoded in LAB, but only ArAT has been experimentally confirmed at present(Zelante et al., 2013; Cervantes-Barragan et al., 2017; Montgomery et al., 2022). It is widely present in *L. reuteri* and has numerous gene copies. The likelihood that LAB also codes for other tryptophan-metabolizing enzymes, in addition to ArAT, depends on the homology evidence of the corresponding enzymes from other bacteria. In this section, we widened the range of reference sequences among species and genera and carried out a homologous search for sequences in the LAB genome. LAB contained a large number of tryptophan metabolism genes according to the results of gene counting. According to the gene annotation results of the sequence with the highest homology, whether most tryptophan metabolizing enzymes can be detected from the genome is species-specific. All LAB had the metabolic enzymes *amiE* and ALD, but not all LAB had their upstream tryptophan metabolism genes encoded. ALD and *amiE*, however, are not indole derivative-specific catalytic proteins (Racker, 1949; Bray et al., 1950), they have additional substrates in other metabolic pathways, indicating that they have additional roles in LAB.

In metabolomics, although we did not detect IPYA in the fermentation broth, as was also the case in earlier studies, it is the only starting point of the ILA metabolic pathway, and the existence of ILA could prove that IPYA appears briefly *in vitro* fermentation. The metabolic level of ILA remained highly species-specific. Although the production of ILA in different *L. salavarius* strains fluctuated greatly, their levels were still significantly higher than those of other LAB strains. Especially FWXBH185 and FBJSY202, which could produce large amounts of ILA, may have great potential to maintain human immune balance (Laursen et al., 2021). IAA and IAM metabolism in LAB deviated from gene predictions, and there was strain specificity. Although we predicted some strains would produce IAM, we were unable to locate any pertinent evidence during the *in vitro* fermentation. IAA also has annotation genes but no phenotype. These results might indicate that IAM and IAA were used as intermediate metabolites in the fermentation process, especially IAM, which was the first step metabolite. As the precursor of IAld (De Mello et al., 1980), IAA might also be difficult to enrich during fermentation. The metabolism of TA and IA in LAB was species-specific, and there was no homologous gene in the LAB genome, but some species produced these metabolites during fermentation. This demonstrates that the availability of some tryptophan metabolic genes in existing open databases is still limited and that to increase the hit rate of the query sequence, the reference sequence range needs to be expanded (Rath et al., 2018). However, although there were deviations between the predicted results of some genes and the phenotype, the average accuracy rate was more than 88%, which indicated that genes could predict the metabolites of LAB to a certain extent. In addition, we tracked the metabolism of IAld in LAB. It can activate the AhR receptor and regulate the level of IL-22 in cells to alleviate the symptoms of host colitis (Renga et al., 2022), which plays an important role in the human immune system. *L. reuteri* DYNDL8M31, FSCPS76L4 and DYNDL2M15 metabolized a large amount of IAld, and these strains might become the focus of probiotic development in the future. The results of metabolomics also revealed the regulation of tryptophan metabolism in LAB of the same species but from different sources. Different sources of separation did not appear to alter the types of tryptophan metabolites that LAB produce, but they may have an impact on some LAB’ metabolic ability. It is important to note that despite the fact that the Mann-Whitney test revealed a significant difference between the production of IAld by *L. reuteri* from fermented rice milk and other strains, due to the small number of samples, this statistical analysis may be influenced by the results of DYNDL8M31, which could produce a high yield of IAld. In general, the types of indole derivatives produced by LAB are not affected by the isolation source, but the relationship between the isolation source and the production of tryptophan metabolites by LAB needs to be explained through more experimental data. Additionally, the growth environment of bacteria has a direct impact on their metabolism of tryptophan. According to earlier research, *Bifidobacterium* could only convert tryptophan to ILA in MRS (Laursen et al., 2021). However, in M9 medium, which has much fewer nutrients than MRS, *Bifidobacterium* can produce a variety of indole derivatives (Fang et al., 2022). This indicates that the various nutrients that are available to bacteria in the intricate environment of the human intestine will also influence their catabolism. There are many nutrients in the proximal colon. All microorganisms are under pressure to grow quickly in order to occupy a more favorable niche. In the proximal colon, microorganisms’ primary mode of catabolism is glycolysis (Roager et al., 2016). In the distal colon, carbohydrate is gradually consumed completely, and protein becomes the main substrate of bacterial catabolism. The previous study demonstrated the distal colon had more than four times the concentration of phenolic compounds produced by the breakdown of aromatic amino acids than the proximal colon (Smith and Macfarlane, 1996). These findings suggest that the resting cell culture approach employed in this study might be more effective than the nutrient-rich fermentation system for producing these tryptophan metabolites. However, bacterial tryptophan metabolism does not only occur in the colon; for example, *Lactobacillus johnsonii* could also decompose tryptophan in the stomach of mice (Zelante et al., 2013).Therefore, the rule of microbial tryptophan metabolism in the human digestive tract needs to be further explored by more realistic simulated intestinal experiments. We attempted to investigate how genes affect the generation of LAB tryptophan metabolites. In the correlation analysis between the number of genes and the yield, we discovered that IAld was significantly negatively correlated with the upstream enzyme *amiE*, which was responsible for catalyzing the production of IAA. IAld production was significantly positively correlated with ILA production in the intra-group correlation analysis (**Figure S3**). This suggests that there might be multiple metabolic pathways for IAld. There is still no experimental proof explaining the precise chemical changes and corresponding catalytic enzyme system in the process of IAA conversion to IAld, even though many studies believe that IAA is the precursor to IAld (Montgomery et al., 2022). The upstream products of IAld and its corresponding metabolic pathway still need experimental proof. The number of ILA and ArAT in LAB was positively correlated, but its Spearman’s ρ was only 0.31, which was a relatively weak correlation. Compared with *Limosillactobacillus* species, *L. salivarius* does not have a large amount of ArAT enrichment. However, its ILA metabolic yield was the highest among all LAB species. This suggested that while ArAT might control ILA production, it was not the crucial factor. Previous studies have proven that both LDH and *fldH* control the production of ILA (Dodd et al., 2017; Laursen et al., 2021). We therefore thought that the homologous protein of LDH and *fldH* may be the enzyme that regulates the high production of ILA in *L. salivarius*. In the phylogenetic analysis of *fldH* homologous proteins, we discovered that three types of *fldH* sequences (type 1-3 *fldH*) of *L. salivarius* were clustered separately and maintained a relatively large genetic distance from the *fldH* of other LAB. Phylogenetic analysis of LDH did not present a similar phenomenon. The metabolic enzymes in the same cluster are thought to have similar roles and catalytic capabilities in phylogenetic analysis, according to earlier research (Laursen et al., 2021). Then, we built protein structures of four types *fldH* of *L. salivarius* FWXBH185, which were used for protein sequence and structural alignment. We discovered that the reference *fldH* and type 1-3 *fldH* protein models can overlap well and both have high TM-scores and low RMSD. This indicates that they might have similar catalytic centers and perform similar functions as reference *fldH*, so that IPYA can be efficiently converted into ILA. However, although type 3 *fldH* had a relatively long genetic distance from all other *fldH* of LAB and had a similar structure with reference *fldH*, it could only be found in 7 *L. salivarius* strains. Since all *L. salivarius* can produce a large number of ILA, this may indicate that type 3 *fldH* is not the enzyme that mainly regulates the level of ILA metabolism in *L. salivarius*. Therefore, We speculate that type 1 and type 2 *fldH* may lead to the ability of *L.salivarius* to metabolize ILA at a high level. The precise function of these two types of proteins in the ILA pathway needs to be demonstrated through experiments in the future.

In conclusion, we emphasize the necessity of using multi-omics to sort out the complex tryptophan metabolism. We discovered through metabolomics that, in addition to IAA, the other tryptophan metabolites of LAB have high species specificity, the metabolic concentration of most strains within a species remains constant and only a small number of strains have strain specificity. Combined with genomic analysis, it was revealed that the tryptophan metabolites of LAB were predictable, and the number of genes and special metabolic genes could regulate the tryptophan metabolism of LAB. These findings offer novel perspectives and experimental evidence that will help the development of probiotics that produce particular tryptophan metabolites.

## Data availability statement

The datasets presented in this study can be found in online repositories. The names of the repository/repositories and accession number(s) can be found in the article/**Supplementary Material**.

## Author contributions

Specific author contributions: TP: investigation, data analysis, writing-original draft, visualization, and methodology; ZP: methodology; ZF: investigation; HW: validation and visualization; JZhu: data analysis; ZH: validation, resources; JZha: funding acquisition and resources; WC: resources, funding acquisition, validation, and data curation; WL: supervision, writing–review and editing, project administration, and funding acquisition. All authors contributed to the article and approved the submitted version.
