# Supplementary material for: Uncovering the specificity and predictability of tryptophan metabolism in lactic acid bacteria with genomics and metabolomics
Source: Front Cell Infect Microbiol. 2023 Mar 13;13:1154346. doi: 10.3389/fcimb.2023.1154346 (PMC10040830; doi:10.3389/fcimb.2023.1154346)
Supplement: Supplementary file 1 [file DataSheet_1.docx]

Supplementary Material

Uncovering the specificity and predictability of tryptophan metabolism in lactic acid bacteria with genomics and metabolomics

**Tong Pan^1,2^, Zhangming Pei^1,2^, Zhifeng Fang^3^, Hongchao Wang^1,2^, Jinlin Zhu^1,2^, Hao Zhang^1,2,4,6^, Jianxin Zhao^1,2,6^, Wei Chen^1,2,4^ and Wenwei Lu^1,2,4,5,6*^**

*** Correspondence:**

Wenwei Lu

[luwenwei@jiangnan.edu.cn](mailto:luwenwei@jiangnan.edu.cn)

## Supplementary Figures


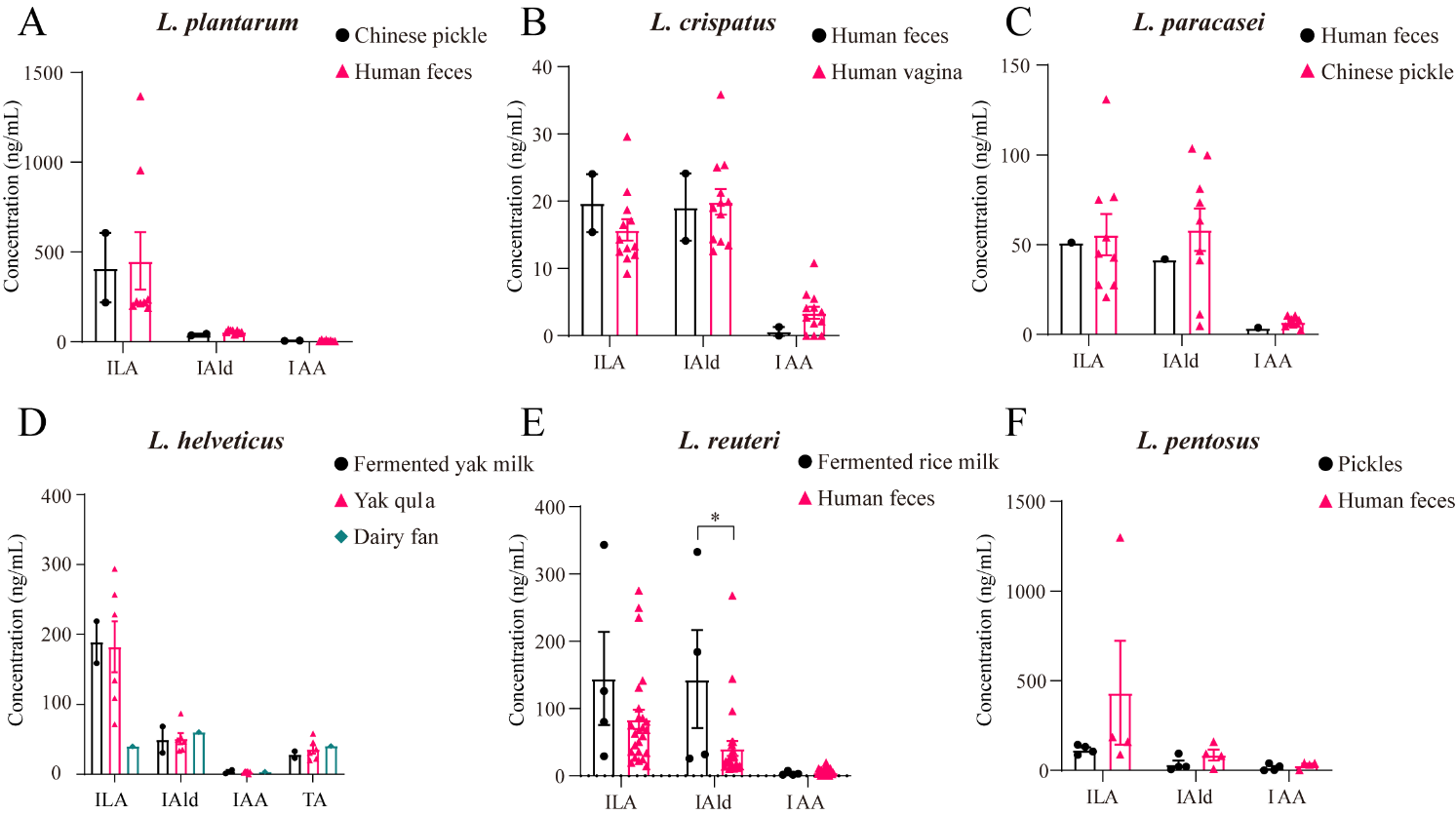


**Figure S1** The results of metabolomics were used to analyze whether there were differences in tryptophan metabolism among strains from different sources. According to the isolation source, LAB were divided into different groups. The Mann-Whitney test was used for significance analysis (n > 1). We also showed the groups (n = 1) that could not be tested for significance. *p < 0.05.


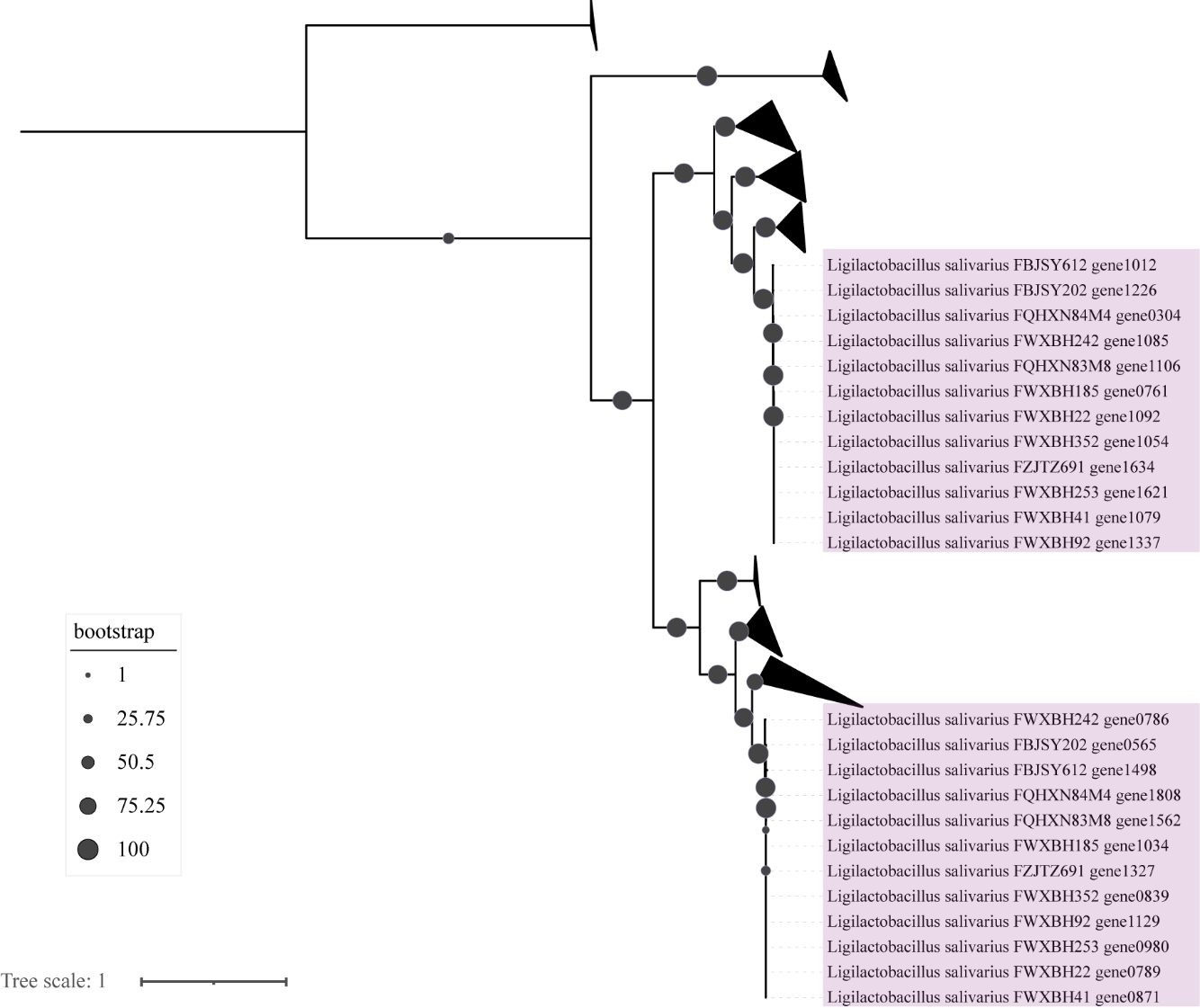


**Figure S2** All lactate dehydrogenase (LDH) genes in lactic acid bacteria (LAB) were used to construct phylogenetic trees, and LDH in *L. salivarius* is specially marked. Some branches of the phylogenetic tree are folded, but this does not affect its topological structure. This is to better show the evolutionary distance between LDH genes from *L. salivarius* and those from other LAB.


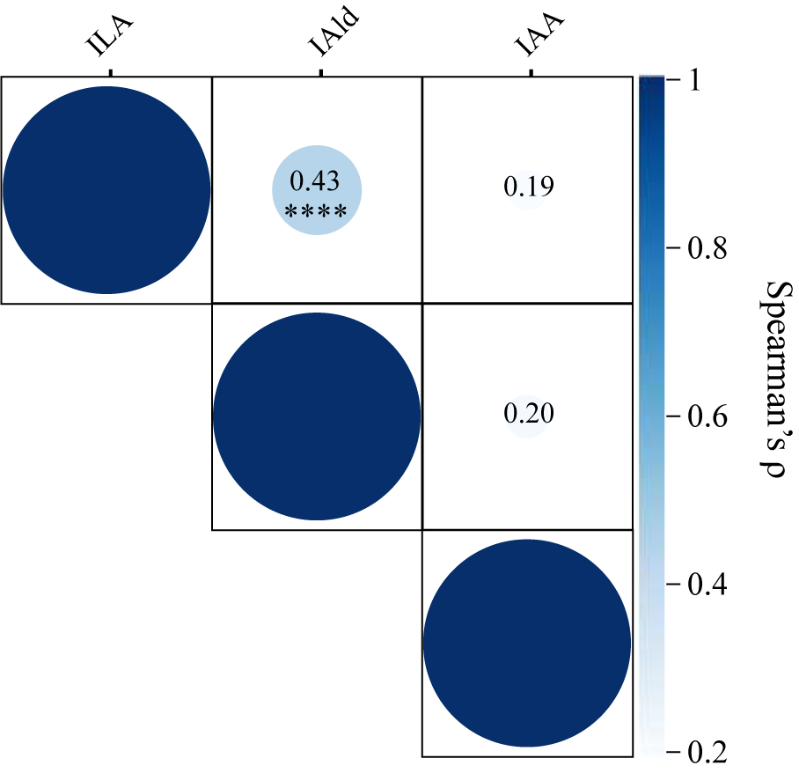


**Figure S3** The correlation between the main tryptophan metabolites of lactic acid bacteria is expressed by Spearman’s ρ, and it will be significant when the coefficient is greater than 0.3. Since both 3-indioleacrylic acid and tryptamine can only be produced by one species, their impact on other metabolites cannot be considered. ****p < 0.0001.
