# Supplementary material for: Uncovering the specificity and predictability of tryptophan metabolism in lactic acid bacteria with genomics and metabolomics
Source: Front Cell Infect Microbiol. 2023 Mar 13;13:1154346. doi: 10.3389/fcimb.2023.1154346 (PMC10040830; doi:10.3389/fcimb.2023.1154346)
Supplement: Supplementary file 3 [file DataSheet_3.docx]

Supplementary Material

Uncovering the specificity and predictability of tryptophan metabolism in lactic acid bacteria with genomics and metabolomics

**Tong Pan^1,2^, Zhangming Pei^1,2^, Zhifeng Fang^3^, Hongchao Wang^1,2^, Jinlin Zhu^1,2^, Hao Zhang^1,2,4,6^, Jianxin Zhao^1,2,6^, Wei Chen^1,2,4^ and Wenwei Lu^1,2,4,5,6*^**

*** Correspondence:**

Wenwei Lu

[luwenwei@jiangnan.edu.cn](mailto:luwenwei@jiangnan.edu.cn)

**Phylogenetic treefile**

(Lacticaseibacillus_rhamnosus_FQHXN3M6_S57_GM002445:0.0000000000,(((Lacticaseibacillus_rhamnosus_FWXBH7L3_S54_GM002447:0.0328245214,((((((Lactiplantibacillus_plantarum_VSCDJY5L5_T20_GM003112:0.0140764241,((Lactobacillus_helveticus_D7S160_S10_GM001941:0.0000010000,Lactobacillus_helveticus_DSCAB10M13_S5_GM001995:0.0000010000)100:0.0037109888,((((Lactobacillus_helveticus_D4A98_S9_GM001398:0.0000000000,Lactobacillus_helveticus_DSCAB9M6_S4_GM001472:0.0000000000):0.0000000000,Lactobacillus_helveticus_DSCAB7M1_S3_GM001756:0.0000000000):0.0000010000,Lactobacillus_helveticus_DSCAB4M1_S2_GM001563:0.0000010000)71:0.0000010000,Lactobacillus_helveticus_DSCAB11L3_S6_GM001973:0.0037109280)41:0.0000023018)100:0.0112175960)56:0.0000020168,Lacticaseibacillus_paracasei_FQHXN98L4_T102_GM003082:0.0035044122)95:0.0034899323,(Lactobacillus_helveticus_D7S160_S10_GM000662:0.0000010000,Lactobacillus_helveticus_DSCAB10M13_S5_GM000629:0.0000010000)52:0.0000027097)88:0.0031486516,Lactiplantibacillus_plantarum_DHuNHHMY2L1_T22_GM002964:0.0038715851)100:0.2025923061,(((((((L.salivarius_3463.081_gene0170:0.0000000000,L.salivarius_1715.381_gene0172:0.0000000000):0.0000000000,L.salivarius_1776.574_gene0169:0.0000000000):0.0000000000,L.salivarius_1479.86_gene0587:0.0000000000):0.0000010000,L.salivarius_3864.553_gene0528:0.0000010000)47:0.0000029955,(L.salivarius_1688.951_gene0136:0.0034756761,(L.salivarius_1625.606_gene0432:0.0000021918,L.salivarius_1588.98_gene0283:0.0000025387)100:0.0040422363)76:0.0000023556)100:0.0174729866,(L.salivarius_1905.057_gene0305:0.0000028089,L.salivarius_1378.367_gene0619:0.0000023450)100:0.0105691957)100:0.0070463826,(L.salivarius_1831.794_gene0088:0.0000010000,L.salivarius_566.553_gene0158:0.0000010000)100:0.0105378538)100:0.1975438184)100:0.2024696555,((((((Lacticaseibacillus_rhamnosus_FQHXN3M6_S57_GM001365:0.0000000000,Lacticaseibacillus_rhamnosus_FXJSW6L1_S51_GM001351:0.0000000000):0.0000000000,Lacticaseibacillus_rhamnosus_FXJSW24L2_S55_GM001278:0.0000000000):0.0000000000,Lacticaseibacillus_rhamnosus_FXJWS10M1_S59_GM001351:0.0000000000):0.0000010000,Lacticaseibacillus_rhamnosus_FWXBH7L3_S54_GM001365:0.0000010000)67:0.0000021398,Lacticaseibacillus_rhamnosus_FZJTZ46M6_S62_GM002462:0.0066894513)100:1.2143869330,((((Lactobacillus_acidophilus_FCQHC4LH1_gene0338:0.0000029141,(Lactobacillus_acidophilus_FGSYC48L79_gene1290:0.0000028811,((Lactobacillus_acidophilus_FHNXY41L162_gene1444:0.0000000000,Lactobacillus_acidophilus_FZJTZ18L25_gene1129:0.0000000000):0.0000028127,Lactobacillus_acidophilus_FSHXBX32L130_gene1398:0.0000010000)83:0.0033920822)66:0.0033967905)50:0.0034006766,((Lactobacillus_acidophilus_FNMGHHT12L40_gene0360:0.0000000000,Lactobacillus_acidophilus_JCM1132_gene0352:0.0000000000):0.0000010000,Lactobacillus_acidophilus_FXJSW24L139_gene0312:0.0000010000)68:0.0000025662)100:0.8411357933,((((((((Lacticaseibacillus_paracasei_VCQYB7171M7_T95_GM002868:0.0033560033,(Lacticaseibacillus_paracasei_VCQBB4126L6_T100_GM001728:0.0000010000,Lacticaseibacillus_paracasei_VCQQJ4174M3_T96_GM000919:0.0000010000)100:0.0033290639)23:0.0000023829,(Lacticaseibacillus_paracasei_FQHXN98L4_T102_GM000998:0.0000000000,Lacticaseibacillus_paracasei_VCQY0Y1157M2_T97_GM001260:0.0000000000):0.0000010000)5:0.0000010000,Lacticaseibacillus_paracasei_VCQJJ296L4_T129_GM001315:0.0000010000)22:0.0000023896,(Lacticaseibacillus_paracasei_VCQBB3125L12_T99_GM001611:0.0000010000,Lacticaseibacillus_paracasei_VCQWX5L7_gene1363:0.0000010000)100:0.0033311217)47:0.0000022933,(Lacticaseibacillus_paracasei_VCQJLP2131L2_T101_GM002612:0.0000026411,Lacticaseibacillus_paracasei_VCQQJ30173L8_July58_GM001714:0.0000010000)100:0.0033336039)100:0.0659167547,(((((Lacticaseibacillus_rhamnosus_FQHXN3M6_S57_GM002222:0.0000000000,Lacticaseibacillus_rhamnosus_FXJSW6L1_S51_GM002208:0.0000000000):0.0000000000,Lacticaseibacillus_rhamnosus_FXJSW24L2_S55_GM002175:0.0000000000):0.0000000000,Lacticaseibacillus_rhamnosus_FXJWS10M1_S59_GM002174:0.0000000000):0.0000010000,Lacticaseibacillus_rhamnosus_FWXBH7L3_S54_GM002224:0.0000010000)100:0.0043087185,(Lacticaseibacillus_rhamnosus_FZJTZ46M6_S62_GM002003:0.0068419422,((Lacticaseibacillus_rhamnosus_FAHWH30L7_S52_GM001854:0.0000000000,Lacticaseibacillus_rhamnosus_2-hydroxyacid_dehydrogenase_family_protein:0.0000000000):0.0000010000,Lacticaseibacillus_rhamnosus_FZJJH6L2_S56_GM001884:0.0000010000)100:0.0034211104)97:0.0025374963)98:0.0841847853)100:0.3960960341,((((Lactiplantibacillus_plantarum_FCQHC24L1_T23_GM001701:0.0000000000,Lactiplantibacillus_plantarum_VSCDJY12L1_T21_GM000840:0.0000000000):0.0000010000,Lactiplantibacillus_plantarum_FCQNA27M4_July14_GM001332:0.0000010000)85:0.0000025345,(Lactiplantibacillus_plantarum_FCQNA28M4_July15_GM000214:0.0032089284,((((Lactiplantibacillus_pentosus_FGDLZ2M10_FGDLZ2M10_GM002131:0.0031985475,Lactiplantibacillus_plantarum_FCQHC25L4_T24_GM001729:0.0032045560)16:0.0000010000,((Lactiplantibacillus_plantarum_DHuNHHMY2L1_T22_GM000931:0.0000000000,Lactiplantibacillus_plantarum_FCQNA29M3_July16_GM000866:0.0000000000):0.0000000000,Lactiplantibacillus_plantarum_VSCDJY5L5_T20_GM001626:0.0000000000):0.0000010000)6:0.0000010000,Lactiplantibacillus_plantarum_FCQNA23M1_July13_GM001262:0.0000010000)8:0.0000010000,Lactiplantibacillus_plantarum_FFJLY44L1_T25_GM000746:0.0031983522)14:0.0000010000)51:0.0031955630)99:0.0867191058,((Lactiplantibacillus_pentosus_DL49_gene0351:0.0032537880,(Lactiplantibacillus_pentosus_RS142_gene0175:0.0032463455,(Lactiplantibacillus_pentosus_L17001_gene0938:0.0000010000,Lactiplantibacillus_pentosus_QSCPS6L1_orf0722:0.0000010000)56:0.0000010000)69:0.0000022799)100:0.0163612750,(Lactiplantibacillus_pentosus_HY311_gene3724:0.0065131233,(Lactiplantibacillus_pentosus_FHuNHHMY67L3_T27_GM001012:0.0032411571,Lactiplantibacillus_pentosus_FZJHZD217_orf1449:0.0000010000)70:0.0000025247)100:0.0034789846)100:0.0837825425)100:0.3055063969)89:0.1082136373,((((((((Limosilactobacillus_fermentum_FJXSRPY6L1_gene1877:0.0000000000,Limosilactobacillus_fermentum_FZJTZ24M3_gene1958:0.0000000000):0.0000000000,Limosilactobacillus_fermentum_FZJTZ23M8_gene1827:0.0000000000):0.0000000000,Limosilactobacillus_fermentum_FZJTZ20M5_gene1791:0.0000000000):0.0000000000,Limosilactobacillus_fermentum_FZJTZ22M6_gene1831:0.0000000000):0.0000000000,Limosilactobacillus_fermentum_FXJSW71_F31_GM002044:0.0000000000):0.0000010000,Limosilactobacillus_fermentum_FXJCJ26M3_gene1837:0.0000010000)53:0.0000010000,Limosilactobacillus_fermentum_FZJTZ2M5_gene1777:0.0034049002)99:0.0046136487,(Limosilactobacillus_fermentum_FZJTZ13M9_gene0526:0.0069111087,Limosilactobacillus_fermentum_FZJTZ25M1_gene1851:0.0000010000)95:0.0022293647)100:0.3719394620)100:0.6802964998)98:0.1812975164,(((((Latilactobacillus_curvatus_FQSYC5M8_gene0793:0.0000010000,Latilactobacillus_curvatus_QGSYC5L4_gene0110:0.0000010000)100:0.0031531858,Latilactobacillus_curvatus_FQHXN80M2_gene0900:0.0031668555)96:0.0063437360,Latilactobacillus_curvatus_FQHXN74M1_gene1386:0.0000022189)100:1.1212423700,((L.salivarius_1831.794_gene1265:0.0033675363,((L.salivarius_1905.057_gene1408:0.0000026903,L.salivarius_1378.367_gene1624:0.0000021421)100:0.0033726628,((((((((L.salivarius_3463.081_gene1804:0.0000000000,L.salivarius_1715.381_gene1221:0.0000000000):0.0000000000,L.salivarius_1776.574_gene0772:0.0000000000):0.0000000000,L.salivarius_1588.98_gene0925:0.0000000000):0.0000000000,L.salivarius_1479.86_gene0658:0.0000000000):0.0000000000,L.salivarius_1688.951_gene0825:0.0000000000):0.0000000000,L.salivarius_1625.606_gene0696:0.0000000000):0.0000000000,L.salivarius_566.553_gene1514:0.0000000000):0.0000010000,L.salivarius_3864.553_gene0864:0.0000010000)39:0.0000025643)56:0.0000027602)100:0.9074336466,(Lacticaseibacillus_rhamnosus_D-2-hydroxyisocaproate_dehydrogenase:0.8691157282,(((((((((((((((((Lactobacillus_helveticus_D4A98_S9_GM000945:0.0000000000,Lactobacillus_helveticus_DSCAB4M1_S2_GM001856:0.0000000000):0.0000000000,Lactobacillus_helveticus_DSCAB11L3_S6_GM000812:0.0000000000):0.0000000000,Lactobacillus_helveticus_DSCAB13M2_S7_GM001024:0.0000000000):0.0000000000,Lactobacillus_helveticus_DSCAB7M1_S3_GM000949:0.0000000000):0.0000000000,Lactobacillus_helveticus_DSCAB10M13_S5_GM001952:0.0000000000):0.0000000000,Lactobacillus_helveticus_DSCAB9M6_S4_GM002079:0.0000000000):0.0000010000,Lactobacillus_helveticus_D7S160_S10_GM001910:0.0000010000)48:0.0000027527,Lactobacillus_helveticus_DYNDL451_S14_GM001486:0.0065773791)100:0.0223668063,((((((Lactobacillus_gasseri_FFJND16L4_gene0607:0.0000000000,Lactobacillus_gasseri_NMGHHHT1L2_gene0699:0.0000000000):0.0000000000,Lactobacillus_gasseri_JSSZ15_gene1409:0.0000000000):0.0000000000,Lactobacillus_gasseri_JSCZD2L1_gene1449:0.0000000000):0.0000000000,Lactobacillus_gasseri_ZJHZD1M5_gene1744:0.0000000000):0.0000000000,Lactobacillus_gasseri_JSWX33L2_gene1846:0.0000000000):0.0000010000,Lactobacillus_gasseri_JCM11657_gene0404:0.0000010000)100:0.0178619006)88:0.0067291376,(((((Lactobacillus_acidophilus_FCQHC4LH1_gene1483:0.0000000000,Lactobacillus_acidophilus_FXJSW24L139_gene1962:0.0000000000):0.0000000000,Lactobacillus_acidophilus_FNMGHHT12L40_gene1544:0.0000000000):0.0000000000,Lactobacillus_acidophilus_JCM1132_gene1548:0.0000000000):0.0000010000,Lactobacillus_acidophilus_FGSYC48L79_gene1421:0.0000010000)53:0.0000028249,((Lactobacillus_acidophilus_FHNXY41L162_gene1999:0.0000000000,Lactobacillus_acidophilus_FZJTZ18L25_gene1496:0.0000000000):0.0000010000,Lactobacillus_acidophilus_FSHXBX32L130_gene1636:0.0000010000)100:0.0033259415)100:0.0213123502)99:0.0043001577,(((((((((((((Lactobacillus_crispatus_FSCDJY13L2_gene0951:0.0000000000,Lactobacillus_crispatus_QJSWX120M1_gene1045:0.0000000000):0.0000000000,Lactobacillus_crispatus_QJSWX128M5_gene1220:0.0000000000):0.0000000000,Lactobacillus_crispatus_QJSWX150M37_gene0811:0.0000000000):0.0000000000,Lactobacillus_crispatus_QJSWX149M5_gene0958:0.0000000000):0.0000000000,Lactobacillus_crispatus_QJSWX166M13_gene1028:0.0000000000):0.0000000000,Lactobacillus_crispatus_QJSWX148M3_gene0729:0.0000000000):0.0000000000,Lactobacillus_crispatus_QJSWX110M1_gene0690:0.0000000000):0.0000000000,Lactobacillus_crispatus_QJSWX169M3_gene1256:0.0000000000):0.0000000000,Lactobacillus_crispatus_QJSWX113M3_gene0455:0.0000000000):0.0000000000,Lactobacillus_crispatus_QJSWX112M2_gene0765:0.0000000000):0.0000000000,Lactobacillus_crispatus_QJSWX154M12_gene0701:0.0000000000):0.0000000000,Lactobacillus_crispatus_QJSWX109M4_gene0819:0.0000000000):0.0000010000,Lactobacillus_crispatus_FSCDJY7L2_gene0689:0.0000026200)100:0.0046161186)100:0.1403965537,((Limosilactobacillus_fermentum_FZJTZ23M8_gene1927:0.0034561236,((((((((Limosilactobacillus_fermentum_FJXSRPY6L1_gene1481:0.0000000000,Limosilactobacillus_fermentum_FZJTZ2M5_gene0430:0.0000000000):0.0000000000,Limosilactobacillus_fermentum_FZJTZ20M5_gene0643:0.0000000000):0.0000000000,Limosilactobacillus_fermentum_FXJSW71_F31_GM001284:0.0000000000):0.0000000000,Limosilactobacillus_fermentum_FZJTZ22M6_gene1359:0.0000000000):0.0000000000,Limosilactobacillus_fermentum_FZJTZ24M3_gene0717:0.0000000000):0.0000000000,Limosilactobacillus_fermentum_FZJTZ25M1_gene0554:0.0000000000):0.0000010000,Limosilactobacillus_fermentum_FXJSW21M2_gene0497:0.0000010000)44:0.0000010000,Limosilactobacillus_fermentum_FZJTZ13M9_gene1553:0.0033190620)41:0.0000010000)84:0.0033139321,Limosilactobacillus_fermentum_FXJCJ26M3_gene0260:0.0000023432)100:0.1348817729)96:0.1056615365,((((Limosilactobacillus_reuteri_DYNDL42M2_gene1581:0.0000000000,Limosilactobacillus_reuteri_FNXYCHL81L1_gene1758:0.0000000000):0.0000000000,Limosilactobacillus_reuteri_FHNXY67L4_gene0410:0.0000000000):0.0000010000,Limosilactobacillus_reuteri_FSCPS25M1_gene1441:0.0027659085)36:0.0000010000,Limosilactobacillus_reuteri_FGDLZ10M5_gene0196:0.0000010000)99:0.0543297558)54:0.0534117147,((Limosilactobacillus_reuteri_FCQHC27L4_gene0593:0.0000022415,Limosilactobacillus_reuteri_FGSYC5L2_gene0077:0.0000021351)100:0.0142270853,((((((((Limosilactobacillus_reuteri_FQHXN127L1_gene0685:0.0100104093,((Limosilactobacillus_reuteri_DYNDL2M15_gene0855:0.0000000000,Limosilactobacillus_reuteri_DYNDL8M31_gene0806:0.0000000000):0.0000010000,Limosilactobacillus_reuteri_DYNDL56M17_gene0749:0.0000010000)89:0.0033197438)24:0.0000022841,Limosilactobacillus_reuteri_FSH40M1_gene0762:0.0033206430)15:0.0000026204,(Limosilactobacillus_reuteri_FCQHC8L6_gene0298:0.0033143135,(((((Limosilactobacillus_reuteri_FSH14M1_gene0538:0.0000000000,Limosilactobacillus_reuteri_FSH6M1_gene0556:0.0000000000):0.0000000000,Limosilactobacillus_reuteri_FXJKS17M8_gene1791:0.0000000000):0.0000000000,Limosilactobacillus_reuteri_FSH39M2_gene0212:0.0000000000):0.0000000000,Limosilactobacillus_reuteri_FSH36M2_gene0175:0.0000000000):0.0000010000,Limosilactobacillus_reuteri_FSH2M2_gene0208:0.0000010000)52:0.0000010000)100:0.0033243739)73:0.0033269894,Limosilactobacillus_reuteri_FSCPS79L4_gene1832:0.0033222882)9:0.0000020529,(Limosilactobacillus_reuteri_FSCPS76L4_gene0451:0.0000010000,Limosilactobacillus_reuteri_FXJCJ4M2_gene1484:0.0000010000)29:0.0000010000)4:0.0000028697,Limosilactobacillus_reuteri_FGSYC2L3_gene0369:0.0066580180)19:0.0000023830,Limosilactobacillus_reuteri_FNXYCHL79L1_gene0391:0.0066511206)90:0.0101135661,Limosilactobacillus_reuteri_FJSXYWG3L1_gene0319:0.0000027404)82:0.0028938336)99:0.0723616447)50:0.0298380141,Limosilactobacillus_reuteri_FAHBZ3L1_gene1458:0.0366812376)47:0.0400201593,((Limosilactobacillus_reuteri_FHNXY71L8_gene0146:0.0000000000,Limosilactobacillus_reuteri_FSDQZ12L6_gene0060:0.0000000000):0.0000010000,Limosilactobacillus_reuteri_FJLHD58L2_gene0316:0.0000010000)100:0.0570130376)100:0.5121922779,(((((((((Limosilactobacillus_reuteri_DYNDL2M15_gene0443:0.0000000000,Limosilactobacillus_reuteri_DYNDL8M31_gene0356:0.0000000000):0.0000027934,Limosilactobacillus_reuteri_DYNDL56M17_gene0332:0.0000028681)100:0.0201979428,(Limosilactobacillus_reuteri_FGSYC2L3_gene0643:0.0132828037,(((((Limosilactobacillus_reuteri_FGSYC5L2_gene0726:0.0033164820,(Limosilactobacillus_reuteri_FAHBZ3L1_gene0208:0.0033042287,Limosilactobacillus_reuteri_FHNXY67L4_gene0036:0.0000010000)38:0.0000026635)83:0.0066372207,((((Limosilactobacillus_reuteri_FCQHC27L4_gene0093:0.0000000000,Limosilactobacillus_reuteri_FSDQZ12L6_gene0322:0.0000000000):0.0000000000,Limosilactobacillus_reuteri_FJLHD58L2_gene0252:0.0000000000):0.0000010000,Limosilactobacillus_reuteri_FHNXY71L8_gene1455:0.0000010000)30:0.0000010000,((Limosilactobacillus_reuteri_DYNDL42M2_gene1139:0.0000000000,Limosilactobacillus_reuteri_FSCPS25M1_gene0097:0.0000000000):0.0000010000,Limosilactobacillus_reuteri_FGDLZ10M5_gene0115:0.0000010000)45:0.0000010000)9:0.0000027161)12:0.0000024413,Limosilactobacillus_reuteri_FNXYCHL81L1_gene0046:0.0033443076)70:0.0066172899,Limosilactobacillus_reuteri_FNXYCHL79L1_gene1130:0.0000021292)65:0.0033052969,Limosilactobacillus_reuteri_FCQHC8L6_gene0762:0.0000027758)25:0.0000021919)37:0.0000026726)47:0.0066090133,((Limosilactobacillus_reuteri_FQHXN127L1_gene2053:0.0067076061,(Limosilactobacillus_reuteri_FJSXYWG3L1_gene1970:0.0000010000,Limosilactobacillus_reuteri_FXJCJ4M2_gene1504:0.0000010000)98:0.0033379425)47:0.0000023182,Limosilactobacillus_reuteri_FSH40M1_gene0046:0.0000010000)29:0.0000024860)37:0.0033206891,(((Limosilactobacillus_reuteri_FSCPS79L4_gene1087:0.0000000000,Limosilactobacillus_reuteri_FXJKS17M8_gene1142:0.0000000000):0.0000010000,Limosilactobacillus_reuteri_FSH6M1_gene0792:0.0000010000)45:0.0000010000,(((Limosilactobacillus_reuteri_FSH14M1_gene1451:0.0000000000,Limosilactobacillus_reuteri_FSH39M2_gene1272:0.0000000000):0.0000000000,Limosilactobacillus_reuteri_FSH36M2_gene1303:0.0000000000):0.0000010000,Limosilactobacillus_reuteri_FSH2M2_gene1155:0.0000010000)79:0.0000010000)97:0.0032940295)34:0.0033601554,Limosilactobacillus_reuteri_FSCPS76L4_gene0910:0.0000020814)100:0.4839135999,((Limosilactobacillus_mucosae_FGSYC1M1_gene1019:0.0000010000,Limosilactobacillus_mucosae_FGSYC42L4_gene0416:0.0000010000)100:0.0202235817,((Limosilactobacillus_mucosae_FGSYC15L3_gene0291:0.0000010000,Limosilactobacillus_mucosae_FJSNT91_gene0049:0.0000010000)100:0.0031517494,(Limosilactobacillus_mucosae_FGSYC17L3_gene0690:0.0033503259,((((Limosilactobacillus_mucosae_FJSNT152_gene1653:0.0032679736,((Limosilactobacillus_mucosae_FGSYC23L4_gene1819:0.0000010000,Limosilactobacillus_mucosae_FJSNT81_gene1938:0.0000010000)44:0.0000010000,Limosilactobacillus_mucosae_FGSYC90L5_gene1620:0.0032723599)42:0.0000023611)98:0.0032732026,((Limosilactobacillus_mucosae_FGSYC19L1_gene0499:0.0000010000,Limosilactobacillus_mucosae_FGSYC43L5_gene0056:0.0000010000)47:0.0000029730,Limosilactobacillus_mucosae_FJSNT141_gene0375:0.0032659571)100:0.0032755052)3:0.0000023620,Limosilactobacillus_mucosae_FGSYC27L1_gene1690:0.0000010000)30:0.0000025933,Limosilactobacillus_mucosae_FJSNT61_gene1762:0.0032732907)95:0.0098190894)99:0.0169795366)96:0.0246342425)100:0.3596932307)88:0.0712773666,((((Limosilactobacillus_mucosae_FGSYC15L3_gene0292:0.0000000000,Limosilactobacillus_mucosae_FJSNT152_gene1652:0.0000000000):0.0000010000,((((Limosilactobacillus_mucosae_FGSYC19L1_gene0500:0.0000010000,Limosilactobacillus_mucosae_FGSYC43L5_gene0057:0.0000010000)100:0.0032542018,Limosilactobacillus_mucosae_FJSNT141_gene0376:0.0000022429)100:0.0032571384,(Limosilactobacillus_mucosae_FGSYC17L3_gene0689:0.0000028116,(Limosilactobacillus_mucosae_FGSYC42L4_gene0417:0.0032582938,Limosilactobacillus_mucosae_FGSYC1M1_gene1020:0.0000010000)100:0.0032580846)100:0.0131110358)26:0.0000027424,(((Limosilactobacillus_mucosae_FGSYC23L4_gene1818:0.0000000000,Limosilactobacillus_mucosae_FJSNT61_gene1763:0.0000000000):0.0000000000,Limosilactobacillus_mucosae_FJSNT81_gene1937:0.0000000000):0.0000010000,Limosilactobacillus_mucosae_FGSYC27L1_gene1691:0.0000010000)64:0.0000010000)32:0.0000010000)18:0.0000010000,Limosilactobacillus_mucosae_FGSYC90L5_gene1619:0.0000010000)20:0.0000022642,Limosilactobacillus_mucosae_FJSNT91_gene0050:0.0033178403)100:0.2394196581)100:0.1902474089,(((Lactiplantibacillus_pentosus_FZJHZD217_orf1244:0.0000010000,(Lactiplantibacillus_pentosus_HY311_gene2570:0.0000010000,Lactiplantibacillus_pentosus_FHuNHHMY67L3_T27_GM003045:0.0000010000)58:0.0000010000)43:0.0000023952,((((((((((Lactiplantibacillus_plantarum_DHuNHHMY2L1_T22_GM000139:0.0000000000,Lactiplantibacillus_plantarum_VSCDJY5L5_T20_GM000204:0.0000000000):0.0000000000,Lactiplantibacillus_plantarum_FCQHC25L4_T24_GM000768:0.0000000000):0.0000000000,Lactiplantibacillus_plantarum_VSCDJY12L1_T21_GM001080:0.0000000000):0.0000000000,Lactiplantibacillus_plantarum_FFJLY44L1_T25_GM002567:0.0000000000):0.0000000000,Lactiplantibacillus_plantarum_FCQNA29M3_July16_GM002698:0.0000000000):0.0000000000,Lactiplantibacillus_plantarum_FCQNA28M4_July15_GM001978:0.0000000000):0.0000000000,Lactiplantibacillus_plantarum_FCQNA23M1_July13_GM000770:0.0000000000):0.0000000000,Lactiplantibacillus_plantarum_FCQNA27M4_July14_GM002735:0.0000000000):0.0000000000,Lactiplantibacillus_pentosus_FGDLZ2M10_FGDLZ2M10_GM000200:0.0000000000):0.0000010000,Lactiplantibacillus_plantarum_FCQHC24L1_T23_GM000092:0.0000010000)99:0.0032694868)89:0.0033536399,(((Lactiplantibacillus_pentosus_DL49_gene3278:0.0000000000,Lactiplantibacillus_pentosus_QSCPS6L1_orf1344:0.0000000000):0.0000000000,Lactiplantibacillus_pentosus_RS142_gene3432:0.0000000000):0.0000010000,Lactiplantibacillus_pentosus_L17001_gene3633:0.0000010000)63:0.0000027904)100:0.3366987404)94:0.1078591292)95:0.1841946903)97:0.1800956901)68:0.0701232758)92:0.1044229352,((((Limosilactobacillus_fermentum_FZJTZ20M5_gene0888:0.1110216191,(((Limosilactobacillus_fermentum_FZJTZ22M6_gene0664:0.0034376381,Limosilactobacillus_fermentum_FZJTZ23M8_gene0644:0.0000010000)99:0.0034246423,(Limosilactobacillus_fermentum_FXJSW21M2_gene0406:0.0000010000,Limosilactobacillus_fermentum_FZJTZ25M1_gene0750:0.0000010000)97:0.0034275555)16:0.0000024312,((((Limosilactobacillus_fermentum_FJXSRPY6L1_gene0580:0.0000000000,Limosilactobacillus_fermentum_FZJTZ2M5_gene0349:0.0000000000):0.0000000000,Limosilactobacillus_fermentum_FZJTZ24M3_gene1001:0.0000000000):0.0000000000,Limosilactobacillus_fermentum_FXJSW71_F31_GM000705:0.0000000000):0.0000010000,Limosilactobacillus_fermentum_FXJCJ26M3_gene0487:0.0000010000)23:0.0000010000)16:0.0000023265)75:0.0156574981,Limosilactobacillus_fermentum_FZJTZ13M9_gene1624:0.0018097159)100:0.4972907587,((((((((L.salivarius_3864.553_gene1883:0.0000000000,L.salivarius_1479.86_gene0848:0.0000000000):0.0000000000,L.salivarius_1776.574_gene1786:0.0000000000):0.0000000000,L.salivarius_1688.951_gene0734:0.0000000000):0.0000000000,L.salivarius_1588.98_gene0600:0.0000000000):0.0000000000,L.salivarius_1715.381_gene0539:0.0000000000):0.0000010000,L.salivarius_1625.606_gene0645:0.0000010000)100:0.5232293758,((((Limosilactobacillus_mucosae_FJSNT81_gene0726:0.0000010000,((Limosilactobacillus_mucosae_FJSNT61_gene0735:0.0032425959,(((Limosilactobacillus_mucosae_FGSYC1M1_gene0646:0.0000000000,Limosilactobacillus_mucosae_FJSNT152_gene1201:0.0000000000):0.0000000000,Limosilactobacillus_mucosae_FJSNT91_gene0328:0.0000000000):0.0000000000,Limosilactobacillus_mucosae_FGSYC42L4_gene1267:0.0000000000):0.0000025502)29:0.0000010000,Limosilactobacillus_mucosae_FGSYC27L1_gene0542:0.0000010000)84:0.0000021363)100:0.0096449164,((Limosilactobacillus_mucosae_FGSYC23L4_gene0671:0.0000010000,Limosilactobacillus_mucosae_FJSNT141_gene1177:0.0000010000)89:0.0000026611,Limosilactobacillus_mucosae_FGSYC90L5_gene1332:0.0000026419)97:0.0000020507)88:0.0032412048,Limosilactobacillus_mucosae_FGSYC17L3_gene0966:0.0065717255)34:0.0000022002,((Limosilactobacillus_mucosae_FGSYC15L3_gene1477:0.0000000000,Limosilactobacillus_mucosae_FGSYC43L5_gene1494:0.0000000000):0.0000010000,Limosilactobacillus_mucosae_FGSYC19L1_gene0832:0.0000010000)95:0.0032345900)100:0.2912977042)98:0.1004574288,((((Limosilactobacillus_reuteri_FQHXN127L1_gene1093:0.0305064126,(Limosilactobacillus_reuteri_FGSYC2L3_gene0307:0.0164828455,(Limosilactobacillus_reuteri_FNXYCHL81L1_gene1418:0.0033018840,(Limosilactobacillus_reuteri_FAHBZ3L1_gene1263:0.0000010000,Limosilactobacillus_reuteri_FGSYC5L2_gene1661:0.0000010000)100:0.0095394821)56:0.0030735634)94:0.0096854648)93:0.0128286606,(((Limosilactobacillus_reuteri_FJSXYWG3L1_gene0923:0.0131907048,(Limosilactobacillus_reuteri_FSCPS79L4_gene1996:0.0000010000,(Limosilactobacillus_reuteri_FXJKS17M8_gene1344:0.0031151340,((((Limosilactobacillus_reuteri_FSH14M1_gene1492:0.0000000000,Limosilactobacillus_reuteri_FSH39M2_gene0152:0.0000000000):0.0000000000,Limosilactobacillus_reuteri_FXJCJ4M2_gene1192:0.0000000000):0.0000010000,Limosilactobacillus_reuteri_FSH36M2_gene0115:0.0000010000)83:0.0000010000,(Limosilactobacillus_reuteri_FSH2M2_gene1298:0.0031644736,Limosilactobacillus_reuteri_FSH6M1_gene1460:0.0000010000)87:0.0000022141)71:0.0000010000)100:0.0031088008)100:0.0031154802)72:0.0000028291,((Limosilactobacillus_reuteri_FCQHC27L4_gene1822:0.0000010000,((((Limosilactobacillus_reuteri_FSDQZ12L6_gene1618:0.0061899625,Limosilactobacillus_reuteri_FHNXY71L8_gene1734:0.0000010000)86:0.0030969623,Limosilactobacillus_reuteri_FGDLZ10M5_gene1484:0.0030949340)21:0.0000010000,Limosilactobacillus_reuteri_DYNDL42M2_gene0820:0.0000010000)24:0.0000010000,Limosilactobacillus_reuteri_FSCPS25M1_gene1566:0.0061935669)46:0.0062154427)45:0.0031012052,(Limosilactobacillus_reuteri_FHNXY67L4_gene1177:0.0031073886,Limosilactobacillus_reuteri_FJLHD58L2_gene1724:0.0000010000)32:0.0000010000)34:0.0031020975)32:0.0031188457,(Limosilactobacillus_reuteri_FSCPS76L4_gene0507:0.0000010000,Limosilactobacillus_reuteri_FSH40M1_gene1350:0.0066209484)99:0.0094027501)9:0.0000025318)40:0.0062401941,Limosilactobacillus_reuteri_FCQHC8L6_gene0363:0.0062874820)40:0.0027581561,Limosilactobacillus_reuteri_FNXYCHL79L1_gene0601:0.0066252538)100:0.2763644785)99:0.1378650981)98:0.2701018614,(((((Limosilactobacillus_fermentum_FZJTZ13M9_gene0918:0.0101565805,(((Limosilactobacillus_fermentum_FXJSW21M2_gene0776:0.0067612083,Limosilactobacillus_fermentum_FZJTZ22M6_gene0414:0.0000010000)25:0.0000010000,Limosilactobacillus_fermentum_FZJTZ23M8_gene0401:0.0000010000)68:0.0000010000,((((Limosilactobacillus_fermentum_FXJCJ26M3_gene1038:0.0000000000,Limosilactobacillus_fermentum_FZJTZ25M1_gene0667:0.0000000000):0.0000000000,Limosilactobacillus_fermentum_FZJTZ2M5_gene0698:0.0000000000):0.0000010000,Limosilactobacillus_fermentum_FZJTZ24M3_gene0837:0.0000010000)72:0.0000010000,Limosilactobacillus_fermentum_FZJTZ20M5_gene0376:0.0033397897)50:0.0000010000)99:0.0033383430)100:0.0033149019,(Limosilactobacillus_fermentum_FXJSW71_F31_GM000517:0.0033211197,Limosilactobacillus_fermentum_FJXSRPY6L1_gene0628:0.0033177046)72:0.0000028602)100:0.5567679322,((L.salivarius_1831.794_gene0506:0.0170585614,((L.salivarius_566.553_gene1435:0.0068742793,(L.salivarius_1905.057_gene0919:0.0000010000,L.salivarius_1378.367_gene1352:0.0000010000)100:0.0033790242)97:0.0033782565,((((((L.salivarius_3864.553_gene1339:0.0000000000,L.salivarius_1588.98_gene0004:0.0000000000):0.0000000000,L.salivarius_1776.574_gene0977:0.0000000000):0.0000000000,L.salivarius_1479.86_gene1758:0.0000000000):0.0000000000,L.salivarius_1688.951_gene1912:0.0000000000):0.0000010000,L.salivarius_1625.606_gene1547:0.0000010000)65:0.0000010000,L.salivarius_1715.381_gene1762:0.0033845858)100:0.0033700058)68:0.0000027529)99:0.0082134519,L.salivarius_3463.081_gene1522:0.0053596344)100:0.4200477767)86:0.0787131966,(((((((Lactiplantibacillus_plantarum_FCQHC24L1_T23_GM001621:0.0000010000,Lactiplantibacillus_plantarum_FCQNA27M4_July14_GM001413:0.0000010000)74:0.0000010000,(Lactiplantibacillus_plantarum_FCQNA23M1_July13_GM001342:0.0031784618,((Lactiplantibacillus_plantarum_FCQNA28M4_July15_GM000296:0.0031893216,((((Lactiplantibacillus_plantarum_DHuNHHMY2L1_T22_GM001014:0.0000000000,Lactiplantibacillus_plantarum_VSCDJY5L5_T20_GM001706:0.0000000000):0.0000000000,Lactiplantibacillus_plantarum_FFJLY44L1_T25_GM002663:0.0000000000):0.0000000000,Lactiplantibacillus_plantarum_FCQNA29M3_July16_GM000945:0.0000000000):0.0000010000,Lactiplantibacillus_plantarum_FCQHC25L4_T24_GM001646:0.0000010000)40:0.0000010000)20:0.0000010000,Lactiplantibacillus_pentosus_FGDLZ2M10_FGDLZ2M10_GM002049:0.0031759196)28:0.0000025713)87:0.0031748671)56:0.0000025741,Lactiplantibacillus_plantarum_VSCDJY12L1_T21_GM000925:0.0031714310)99:0.0644121189,((Lactiplantibacillus_pentosus_DL49_gene0266:0.0000010000,Lactiplantibacillus_pentosus_L17001_gene0846:0.0000010000)83:0.0000010000,(Lactiplantibacillus_pentosus_QSCPS6L1_orf2774:0.0000010000,Lactiplantibacillus_pentosus_RS142_gene0259:0.0000010000)49:0.0000021261)99:0.0061727675)63:0.0002491089,Lactiplantibacillus_pentosus_HY311_gene0479:0.0000010000)68:0.0031968278,Lactiplantibacillus_pentosus_FZJHZD217_orf1531:0.0032341431)81:0.0097036316,Lactiplantibacillus_pentosus_FHuNHHMY67L3_T27_GM001092:0.0000020097)100:0.3149975858)100:0.1454832327,(((Lacticaseibacillus_paracasei_VCQJLP2131L2_T101_GM001307:0.0036900098,Lacticaseibacillus_paracasei_VCQQJ30173L8_July58_GM001897:0.0000010000)79:0.0000022187,((Lacticaseibacillus_paracasei_FQHXN98L4_T102_GM001458:0.0066262427,(Lacticaseibacillus_paracasei_VCQBB4126L6_T100_GM000606:0.0000010000,Lacticaseibacillus_paracasei_VCQQJ4174M3_T96_GM001043:0.0000010000)100:0.0033013979)85:0.0032987535,(((Lacticaseibacillus_paracasei_VCQBB3125L12_T99_GM002641:0.0000000000,Lacticaseibacillus_paracasei_VCQWX5L7_gene2624:0.0000000000):0.0000010000,Lacticaseibacillus_paracasei_VCQJJ296L4_T129_GM001687:0.0000010000)72:0.0000010000,(Lacticaseibacillus_paracasei_VCQY0Y1157M2_T97_GM000202:0.0032898130,Lacticaseibacillus_paracasei_VCQYB7171M7_T95_GM000973:0.0065614453)82:0.0032897705)64:0.0000010000)91:0.0099251486)100:0.0780815211,(Lacticaseibacillus_rhamnosus_FAHWH30L7_S52_GM001145:0.0150622145,(((((Lacticaseibacillus_rhamnosus_FQHXN3M6_S57_GM000245:0.0000000000,Lacticaseibacillus_rhamnosus_FXJSW24L2_S55_GM000091:0.0000000000):0.0000000000,Lacticaseibacillus_rhamnosus_FXJSW6L1_S51_GM000245:0.0000000000):0.0000000000,Lacticaseibacillus_rhamnosus_FXJWS10M1_S59_GM000091:0.0000000000):0.0000010000,Lacticaseibacillus_rhamnosus_FWXBH7L3_S54_GM000091:0.0000023171)100:0.0031829350,((Lacticaseibacillus_rhamnosus_FZJJH6L2_S56_GM002773:0.0000010000,Lacticaseibacillus_rhamnosus_FZJTZ46M6_S62_GM001497:0.0000010000)54:0.0000025883,Lacticaseibacillus_rhamnosus_D-2-hydroxyacid_dehydrogenase:0.0000010000)100:0.0065054608)99:0.0045949258)100:0.1382673196)100:0.4162672645)97:0.1941214197)94:0.1585638528)100:0.8104113311)81:0.1389466201)100:0.8469695873)100:0.2855343981)61:0.0000021587,Lacticaseibacillus_rhamnosus_FXJSW24L2_S55_GM002486:0.0000010000):0.0000010000,Lacticaseibacillus_rhamnosus_FXJWS10M1_S59_GM002485:0.0000000000):0.0000000000,Lacticaseibacillus_rhamnosus_FXJSW6L1_S51_GM002431:0.0000000000);
